# The experience of albinism in France: a qualitative study on dyads of parents and their adult child with albinism

**DOI:** 10.1186/s12916-024-03251-z

**Published:** 2024-01-29

**Authors:** Hugo Fournier, Marie Hasdenteufel, Constance Garrouteigt, Mathieu Perie, Antoine Gliksohn, Béatrice Jouanne, Smail Hadj-Rabia, Benoit Arveiler, Fanny Morice-Picard, Bruno Quintard

**Affiliations:** 1 Laboratory of Psychology (LabPsy) UR 4139, University of Bordeaux, Bordeaux, F-33000 France; 2 National Institute for Research in Digital Science and Technology, Talence, F-33405 France; 3 Genespoir Association, Rennes, F-35000 France; 4 Global Albinism Alliance, Fontenay-sous-Bois, F-94120 France; 5 APHP Paris Necker-Enfants Malades, Paris, F-75015 France; 6 CHU Bordeaux, Bordeaux, F-33404 France; 7 Laboratory of Rare Diseases: Genetics and Metabolism (MRGM) INSERM U1211, University of Bordeaux, Bordeaux, F-33076 France

**Keywords:** Albinism, Rare diseases, Dyadic functioning, Disability adjustment, Visual impairment, Daily life, Systemic approach, Qualitative research

## Abstract

**Background:**

To date, almost no research on the psychosocial implications of albinism has been conducted in France and an exploration of albinism-related experiences could be beneficial, in order to better understand this condition. The aim of this study was to examine how French people with albinism and their parents live with and adapt to this condition in all the areas of their lives.

**Methods:**

Semi-structured phone interviews were conducted with 9 parent-child dyads, each participating separately. Participants were recruited by convenience sampling, thanks to the combined efforts of a patient association (Genespoir) and professionals from the partner medical referral centers involved in the project. Dyads in which the individual with albinism had any comorbidity were excluded. The interviews were then transcribed and subjected to in-depth thematic analysis. Two codebooks were constructed in a mirrored process: one for people with albinism; the other for their parents. They were finally merged at the end of the coding step.

**Results:**

Four main categories were identified: personal perceptions and social representations of albinism, difficulties and obstacles encountered by people with albinism, resources and facilitators, and the importance of parent-child functioning. The results indicated that experiences of stigmatization during childhood and adolescence are common and that people with albinism face challenges in adapting to certain obstacles related to their visual impairments (VI) (e.g., inability to drive a car; eye strain...). Parents emerged as one, if not as the main, source of support for people with albinism throughout their development. Although external support systems exist to assist them in various aspects of their lives, some of them primarily rely on their own personal resources to cope.

**Conclusions:**

This research highlights the importance of a systemic and transdisciplinary approach to make sure families receive the support that best meets their needs.

**Supplementary Information:**

The online version contains supplementary material available at 10.1186/s12916-024-03251-z.

## Background

Albinism refers to a group of rare genetic diseases that are broadly characterized by poor vision and a variable hypopigmentation phenotype. The absence or decrease in pigmentation can occur in the skin, hair, and the eyes, known as oculocutaneous albinism, or only impair pigmentation in the eyes, known as ocular albinism. There are also much rarer syndromic forms of albinism, such as Hermansky–Pudlak syndrome and Chediak–Higashi syndrome, characterized by more severe phenotypes that affect a range of additional cell types beyond pigment cells [1]. New genes associated with albinism have recently been discovered, in both oculocutaneous and syndromic forms [2, 3]. Thus, albinism and its many forms have been widely investigated in genetics [4–6], but psychological studies on albinism life experiences are quite limited, having been mainly led on the African continent, and the results are often not generalizable [7]. In addition, many of the papers on albinism are in the form of essays, testimonies, or pleadings [8–12]. On the African continent, persons with albinism (PWAs) are the target of violent discrimination associated with multiple misconceptions and superstitions [13–16], which can affect young PWAs’ relationships with members of their own family [17]. Nevertheless, several studies have also looked at young PWAs’ life experiences in terms of education [18–22], social functioning [23, 24], and self-concept [25] and have concluded that all three of these domains could be largely affected by the stigma that they may experience. The last decade has seen a significant amount of research emerge in health psychology regarding adult with albinism, especially in Africa [26–40].

In France, however, only a few studies in psychology have been conducted on this population, such as the construction of a questionnaire to assess the Burden of Albinism [41] and the development of a therapeutic patient education program for the families of PWAs [42]. The role and implications of families in adjusting to a health concern have been extensively studied in the context of disability or chronic illness [43–45], but there are very few data on the experience of persons with albinism and even less on the experience of their relatives. The family is a complex and open system that aims to maintain a balance between stability and change through continuous co-construction processes among its various members, highlighting the interdependence between the life cycles of the individual and the family [46–48]. Although many studies have investigated overall family functioning in various contexts, the task remains challenging due to the complexity of family dynamics and the environmental factors that influence them [49, 50].

To limit the complexity of such an analysis, some researchers have chosen to focus only on specific family dyads. Bodenmann (1997) [51] has conceptualized dyadic coping as an interactive process between the two members of a dyad aimed at helping each other cope with daily stress, by adopting joint strategies or by providing emotional and practical support to their partner. Several other authors have followed his path by operationalizing the concept through the building of various dyadic coping assessment tools [52, 53] and statistical analyses [54, 55]. Thus, several studies have highlighted the protective nature of this dyadic coping [56, 57], including its favorable impact on the quality of life and stability of each partner [58–60].

To set the research objectives and guide the construction of the interview guides, we drew upon the “Ecological Systems Theory” developed by Bronfenbrenner (1979, 1986) [61, 62]. This theory presents a scientific approach to studying lifespan development, emphasizing the interrelationship of various developmental processes, such as cognitive, social, and biological. Based on this model, we aimed to explore the individual and shared experiences of albinism within parent and adult child with albinism dyads, outlining the following specific objectives:

- Explore the perceptions and understandings of PWAs and their parent about albinism
- Question the perceptions and roles of health professionals, associations, and institutional bodies in France in terms of support and guidance for albinism
- Understand how PWAs have grown up with albinism and identify the challenges associated with each stage of their development
- Examine the daily management strategies employed by PWAs in dealing with their albinism
- Analyze the nature of interactions between PWAs and their parent, focusing on parental contributions to managing the condition

## Methods

This is a qualitative research study based on interviews, with an exploratory (without any a priori research hypotheses), cross-sectional (conducted at a single time point), and retrospective (recalling experiences from various life stages) design.

### Participants

The study included nine parent-child dyads who met the inclusion criteria. In order to participate in the study, participants had to have albinism, regardless of its form, and be over 18 years old and have no mental or physical comorbidities. PWAs were also required to participate with one of their parents (also without any physical or mental comorbidities). Participants were selected through convenience sampling as part of a larger mixed study (ALBIPSY). The research was disseminated by various means in order to maximize our chances of recruiting a sufficient number of dyads (i.e., promoted on the official website of the French Albinism Association Genespoir; posters displayed in specific centers; emailing specific patient lists; presented to patients and families during consultations). Then, participants expressing interest in the study were provided access to an online consent form where they could give their agreement and contact details so that we could get in touch with them. The recruitment process firstly involved contacting the voluntary PWA, who then provided the contact details of their parent after obtaining their consent. The investigator then contacted the parent (via email or phone) to explain the research and confirm their participation. This study did not undergo a process with an ethics committee since it was evaluated by a university committee as being outside the scope of the Jardé law (Additional file 1: NRIPH Attestation). Therefore, to comply with ethical requirements for all research involving human persons, participants were asked to sign a written informed consent form after reading an information sheet outlining the study’s objectives, issues, and procedures, their rights to withdraw their participation, access and amend their data, and the guarantee that their anonymity would be preserved (in line with the European General Data Protection Regulation). For the purposes of this study, no participant chose to exercise these rights.

### Procedure

Although the current study was conducted by the University of Bordeaux, the entire research protocol was digitized to overcome constraints related to geographical distance of some participants. The interviews were conducted by the first author (HF) from September 2020 to December 2021, using a semi-structured interview guide. They explored issues of concern and life experiences across different domains, such as education, medical follow-up, daily living, illness representations, relationships with institutions, family and dyadic functioning, social interaction, and stigma and discrimination (Additional file 2: Semi-structured interview guides). The interview guide for the PWAs was constructed first, followed by the parents’ interview guide in a mirrored process.

The interviews were conducted by telephone or videoconference and directly recorded on Audacity open-source software. The average duration of the interviews was 43 min and 4 s (range: 27:10–68:36). The interviews were transcribed manually or assisted by a machine learning model (“Whisper” by OpenAI) with the help of the fourth author (MP) to facilitate the transcription work.

### Data analysis

Data were analyzed using the principles of thematic content analysis [63]. To develop an a priori thematic structure, the first two interviews of each category of participants (parent and PWAs) were analyzed in detail in a double-blind process with the second author (MH). The two analysts then pooled their thematic trees to propose an optimal thematic structure under the supervision of the last author (BQ). The first author then conducted a sequential analysis of the remaining interviews based on this a priori structure.

Following the recommendations of O’Connor and Joffe (2020) [64], intercoder agreement was calculated through recoding by the third author (CG) based on three PWA interviews and two parent interviews. The average Cohen’s Kappa coefficients were 0.91 and 0.78 respectively, indicating a good degree of intercoder agreement for both groups.

Thematic saturation was reached after the ninth dyad was coded and no new significant themes emerged once the sixth interview was coded for each group [65–67]. In view of the exhaustive nature of the topics covered, the synthesis and formatting of the results were discussed by the first and last authors (HF and BQ) to make data presentation as clear and meaningful as possible. To facilitate this process, only themes reported by more than three participants have been presented in the results section. Whenever a theme is predominantly expressed by either PWAs or parents, such distinction will be explicitly specified in the text. Otherwise, it indicates that the theme has been addressed by both to a similar extent.

## Results

The sample consisted of 18 participants (nine adult-children with albinism, nine affiliated parents), with a majority of 14 women and four men (see Table 1). The sample included five mother-daughter dyads, three mother-son dyads, and one father-daughter dyad, with no father-son dyads. The frequency at which the two members of a dyad saw each other varied, with three dyads seeing each other daily, two dyads meeting once or several times a month, and two dyads meeting only during holiday periods, while the two remaining dyads did not specify. The average age of the PWAs was 24,22 years (range: 18–42 years), with many of them being students (*n* = 5/9; 55.56%). The average age of the parents was 53,44 years (range: 41–72 years), with most of them having a professional activity (*n* = 8/9; 88.89%).

**Table 1:** Description of the parent-child dyads included for qualitative analysis

Dyad n°	PWA		Dyad		Parent	
	Age	Gender	Family relationship	Proximity (frequency)	Age	Gender
1	19	F	Daughter-mother	Weekly	52	F
2	22	F	Daughter-father	Daily	49	M
3	18	M	Son-mother	Daily	48	F
4	26	F	Daughter-mother	Quarterly	58	F
5	22	M	Son-mother	*NA*	59	F
6	20	F	Daughter-mother	Daily	46	F
7	18	M	Son-mother	Each school holidays	41	F
8	42	F	Daughter-mother	Monthly	72	F
9	31	F	Daughter-mother	*NA*	56	F

*F*, female; *M*, male

Of the nine PWAs, eight individuals were diagnosed with albinism within the first 6 months after birth, while only one person received a diagnosis at a later age (3 years after birth). Furthermore, five participants underwent molecular diagnosis and thus knew their type of albinism: three had oculocutaneous albinism, of which two were able to specify the type (OCA1), and two had ocular albinism (OA1). Regarding siblings, only two participants had a brother or sister with albinism.

As a result of the thematic content analysis, four main categories were identified in each group: personal perceptions and societal representations of albinism, the main difficulties and obstacles encountered by PWAs, PWAs’ main resources and facilitators, and the role of parent-child functioning (or dyadic functioning) in shaping the experiences of PWAs. Detailed information regarding each of these themes will be provided in the subsequent sections in descending order of citation frequency. All the results presented in these sections only apply to the interviewed PWAs and their parents.

### Personal perceptions and societal representations of albinism

**Fig. 1:**
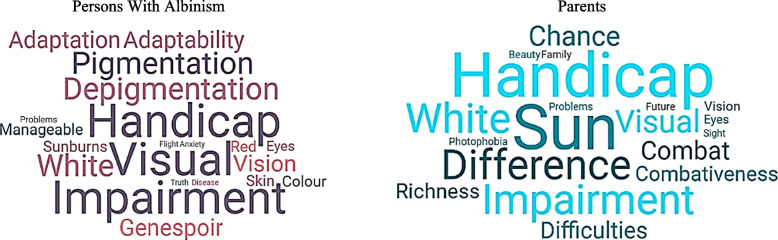
Word clouds about albinism perceptions by PWAs and by parents To read the word cloud: the larger the word, the more frequently it is cited; the shades of color hold no significance; they are solely used to enhance readability; the term “Genespoir” refers to the French Association for Albinism

In order to have an idea of the overall mental image they held of albinism, the first question posed to the participants was “what are the three words that come to mind when you hear the term *albinism*?”. This question is a simple and effective way to access the social representations of individuals within the context of interviews [68]. Upon examining the word clouds presented in Fig. 1, it is obvious that the lexical field of disability and adaptation to disability predominates. PWAs focused on the visual aspect of the condition, while parents highlighted the sensitivity to sunlight. Notably, the notion of “fight” or “fighting spirit” was only present in parents’ representations, suggesting the challenges faced by families in living with such a condition. The presence of the term “Genespoir,” the name of the French Association for Albinisms, is also noted.

**Fig. 2:**
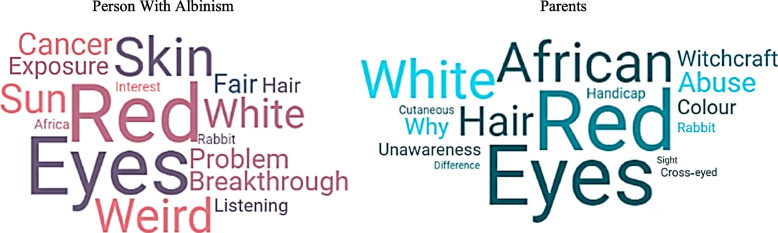
Word clouds about societal representations of albinism by PWAs and their parents To read the word cloud: the larger the word, the more frequently it is cited; the shades of color hold no significance; they are solely used to enhance readability

When asked about albinism from the perspective of someone unfamiliar with it, PWAs and their parents commonly mentioned the “red eyes” characteristic (see Fig. 2). Upon closer examination of the word cloud generated from parents’ responses, terms such as “African” and “witchcraft” also emerged.

#### How do they define albinism?

Participants provided a clear explanation of albinism when asked to explain it in their own words (Additional file 3: Table detailing thematic content analysis). However, not all participants used the same terminology to describe albinism; some used the term “disease” or “disorder,” while others, uncomfortable with this phrasing, preferred to use alternative expressions such as “disability,” “uniqueness,” “condition,” or “affection.” In total, four PWAs and six parents did not consider albinism to be a disease or a disorder. When asked why, they said that, to them, the generic term “disease” or “disorder” implied serious illnesses (e.g., cancer) or viral diseases (e.g., the flu).

#### What do they think about the way PWAs are perceived by the general population?

Concerning the perceptions of others, interviewed participants highlighted the limited awareness, in the general public, of the visual impairment (VI) associated with albinism. Many people do not realize that numerous PWAs may experience a significant VI that includes reduced visual acuity, sensitivity to the light (photophobia), and poor depth perception. Thus, many people do not realize that albinism can cause different forms of disability. Additionally, the general public’s limited knowledge or understanding of albinism can be attributed, in part, to its rarity.

Stereotypical beliefs and misconceptions surrounding albinism were also prevalent. Many people associate albinism with animal imagery, such as albino rabbits, or with African mythology and folklore. Other misconceptions are the beliefs that albinism is contagious or that it would necessarily involve blindness, which is untrue. Negative stereotypes and misconceptions are also reinforced by the fact that characters with albinism are depicted in movies and literature as sneaky and untrustworthy.

Finally, parents in particular reported that people often reduce albinism to a physical stereotype, such as whiteness and red eyes, which fails to recognize the complexities of the condition. In addition, albinism is often not that visible in Europe, and this may contribute to a lack of awareness and understanding of the condition.

In this respect, the public’s understanding of albinism seemed to be often limited and sometimes fraught with misconceptions and occasionally stereotypical beliefs.

“When I say that I’m an albino, I say, ‘you know, it’s like white rabbits’—‘Oh yeah, okay,’ and then it makes more sense to them. But, in any case, it’s not at all associated with visual problems, that’s for sure. Generally, people are a bit surprised: ‘Oh really! Albino means they have visual problems?’—generally, they don’t really know. Uh, but I think that, yeah, I really associate it with an imagery also linked to Africa, maybe, where there have been certain representations of albinism that are quite unique.” (PWA1)

### Difficulties and obstacles encountered by PWAs

PWAs often encountered significant difficulties and obstacles throughout their lives, particularly in their relationships with others (Additional file 3: Table detailing thematic content analysis).

#### Relationships with others

One of the main issues reported by PWAs and their parents is that of stigma and discrimination, especially during childhood and adolescence. This could take the form of stigmatization based on appearance, teasing, rejection, and exclusion. Very often, this feeling of stigmatization faded with time, almost disappearing upon reaching adulthood.

A lack of understanding of what albinism entails is a common issue that PWAs face. Parents emphasized the lack of awareness in surrounding individuals regarding VI, while PWAs highlighted the lack of awareness regarding photosensitivity. In this respect, PWAs primarily reported that these negative experiences could lead them to withdraw socially or, in some cases, may have led them to hide their condition from the rest of the world.

“I think middle school was very difficult for her. In terms of social issues, she was rejected by her friends... and she took it very badly. [...] at that time, she was hoping that my husband would be transferred to another region because she was taking it very hard.” (P6^1^)

#### Transport and mobility

In addition to social challenges, PWAs also encountered difficulties with transport and mobility. Many PWAs do not have sufficient visual acuity to drive a car or motorcycle. By law, they are not allowed to get a driver’s license.

Difficulties also arise when navigating public areas as a pedestrian. PWAs, in particular, reported that they may have difficulty reading signage and finding their way, which can increase their likelihood of getting lost.

“I think that when you don’t see well, you get lost all the time: you don’t know where you are, you don’t know on what street you are, you missed the bus stop, you get on the wrong train... And actually, she would call me completely panicked, saying, ‘I don’t know where I am.’” (P1)

#### Complex and frequently unsatisfactory interactions with healthcare providers

It is mainly parents who mentioned the challenges they and their children encountered in their interactions with health professionals. One major issue is the lack of healthcare support provided to parents and PWAs. This can include a lack of information regarding possible aids and procedures as well as an absence of explanations about albinism and its related symptoms, such as VI.

Another problem is that many health care professionals were not familiar with albinism and its impact on daily life. This could lead to an inversion of the patient/expert roles, with the PWA having to educate professionals.

Parents also faced difficulties when doctors communicated a diagnosis, either because these professionals said it insensitively or because they provided inaccurate information (for example, that their child would go blind).

“Actually, it’s true that the doctors told my parents at that time that I wouldn’t be able to pursue my studies, which of course turned out not to be true.” (PWA9)

#### Complex and frequently unsatisfactory interactions with the education system

VI could pose significant challenges for students in the education system. PWAs, in particular, reported that some teachers resisted adjusting to the needs of visually impaired students, while others did not care to make the necessary adjustments. Moreover, some teachers lacked knowledge about VI or felt unable to provide appropriate support.

Reading the blackboard was also a big challenge for participants when they were and this difficulty was increased at university by the greater distance between the students and the whiteboard/screen.

In this context, only parents shared that some schools were reluctant to enroll visually impaired students: some principals refused them because of the potential impact on the school’s achievement statistics. In some cases, principals urged parents to take their visually impaired children to specialized schools.

“I was thinking about a comment—a serious one—that a teacher made when she was in kindergarten. She came to me at the end of the year, she said, ‘You know Mrs. M., you’re going to have to move.’—‘Oh really?’—‘Oh yes, because she’s going to have to go to a special school, you can’t stay in a regular school.’ So that was pretty abrupt, you see. You see, there you go, another comment that really got stuck in my craw. It’s crazy, though, it’s crazy.” (P1)

#### Complex and frequently unsatisfactory interactions with disability services agencies

Flaws in disability services agencies (DSAs) emerged as a prevalent challenge experienced by the families of PWAs, underscored in parental interviews. Administrative procedures were complicated and time-consuming, making it difficult to compile the necessary documentation. There was no support provided in the process of filing for disability benefits, and the DSA was often difficult to contact. These issues could lead PWAs to frustration and discouragement when seeking support.

“I would say around 10 to 12 hours for putting together the application, and you can add an additional four hours for attempting to contact them, and then another four hours for writing letters.” (PWA1)

#### Aspirations and projects undermined

Parents in particular indicated that the condition of PWAs could exert a significant impact on their life aspirations and choices (e.g., military professions). In addition, parents highlighted the challenges encountered by PWAs in commuting to rural work areas, which could affect their housing choices.

“I wanted to join the army, that was my life goal, you could say. I really wanted to, but I can’t. [...] So, I wasn’t feeling very well, yeah... It was a bit of a darker period.” (PWA3)

#### Difficulties in managing somatic disorders

Regarding somatic problems, PWAs and parents reported eye strain and photosensitivity, which can cause fatigue and headaches, particularly when compensating for their VI (and exacerbated by nystagmus). Moreover, photosensitivity can lead to certain places becoming more or less livable depending on the time of the day.

“So, it’s true that daylight can be a bit complicated for me. And then, in addition, we don’t have curtains—well, it’s a bit complicated—but we don’t always have curtains at home, so that’s a bit annoying. There are times of the day when I don’t feel well in certain rooms.” (PWA8)

#### Difficulties in managing the demands of society

The last difficulty we will detail here is that some PWAs reported they constantly had to adapt to fit in with society (this theme also emerged in two parent interviews). This pressure to appear “normal” could lead to a burden where individuals were continuously adapting without speaking up about their own difficulties. Moreover, some individuals said they refused certain aids in order to avoid deviating from the norm, which could further compound the problem. This constant adaptation could also cause others to be unaware of the difficulties PWAs’ face, leading to a lack of understanding and support. This illustrates a central paradox in the experience of PWAs that can be psychologically challenging: the desire to appear normal while presenting an “abnormality.”

“Is it always a good thing for me to be constantly in a state of adaptability and not always talk about the underlying difficulties of my albinism? Because I used to have a very positive outlook where I would keep all the difficult things to myself and wouldn’t talk about them. [...] The real challenge was the way I looked at myself, I think. How capable I believe I am? to what extent I position myself as ‘You can do anything, but at the same time, you can’t do things like everyone else.’? I think that’s really it... yeah.” (PWA1)

### Resources and facilitators

In order to deal with all of these challenges, over the course of their lives, interviewed PWAs have developed a wide variety of coping strategies, which we will detail below (Additional file 3: Table detailing thematic content analysis).

#### Personal resources: coping strategies

##### Mobility strategies

These strategies involved using public transportation, personal transportation alternatives such as bikes, or seeking support from family and friends for car transportation. As pedestrians, PWAs also asked for help from passersby to find their way or used digital tools such as smartphones for wayfinding and orientation.

##### Health-related strategies for physical issues

Another important coping strategy for PWAs was sunlight protection (e.g., sunglasses, highly protective sunscreens…) and tools/techniques that can improve their everyday vision (e.g., glasses for high prescription, smartphone camera zoom function, portable monocular/binoculars…).

##### Relational and cooperative strategies

PWAs often asked others for help to read or see something or learn how to use certain tools. They also found support from friends to cope with stigmatization by explaining their condition to others to avoid misunderstandings or comments.

##### Seeking information about albinism

PWAs mainly reported that seeking information about albinism was vital to effectively manage their condition. They could acquire knowledge through online resources or through albinism associations, with a desire to understand the mechanisms of albinism, for example, through genetics.

##### The need for adaptability at all costs

Finally, the necessity of cultivating adaptability emerged (primarily from PWAs) as a significant mindset. This attitude enabled them to develop all these coping strategies and to forget the challenges they had to face. Thanks to this adaptive force, PWAs were able to accomplish even the most difficult activities, shielding them from a poor quality of life.

“This forced adaptability has a positive side in that I have always adapted and I know how to adapt, and it’s actually complicated to know how to adapt to everything because we don’t have limits to adaptation, we accept everything, we end up being capable of accepting and saying: ‘Ok, I need to adapt? Well, I’ll adapt’.” (PWA8)

#### External resources: support provided in different spheres of life

##### Educational adjustments

School accommodations for individuals with albinism were very important in ensuring they have equal access to education. This included accommodations in the classroom (e.g., sitting in the front row or closer, using a video magnifier, being allowed to move around), during exams (e.g., additional time, separate room, administering the exam on a computer), or in physical education (e.g., adjusted equipment or exemption from evaluation when needed). Generally speaking, parents and PWAs stated that finding mutually amicable arrangements with teachers made learning more manageable.

“Meaning that several students benefited from it [PIU^2^], and we didn’t all necessarily have the same needs. I know that I like Arial 16—for example—and others preferred 18 or 24 or Comic Sans MS—anyway—everyone has their own adaptation. And it’s true that it was quite... that it was quite good to have that.” (PWA9)

##### Support provided by medical and social institutions

Healthcare and social support services are two of the main resources PWAs and their families had to deal with. First of all, ophthalmological surgical interventions that correct nystagmus or strabismus were helpful in reducing VI. Likewise, genetic testing could provide information about the probability of having a child with albinism or could help PWAs to learn more about their own condition, such as the type of albinism.

Parents especially indicated that having skilled medical experts shortly after the birth of a child with albinism allowed prompt diagnosis and immediate referral to appropriate healthcare professionals.

Organizations that support PWAs and their families throughout childhood and adolescence also proved beneficial. Those that work with a transdisciplinary approach could assist PWAs in pursuing regular schooling, offered parental support and guidance, provided psychological support, and helped raise awareness among PWAs’ social circles about their experience.

Institutions could offer significant support by supplying, donating, or financing educational and professional equipment or allowances.

“It’s true that we had good support each time from SAAAS^3^, which allowed her to have a normal education, and that there were facilitators who came to help her and explain what visual impairment was to the school. Because it’s true that it’s a disorder that isn’t well-known, and that teachers aren’t familiar with. That’s why these visual impairment centers are really well-made.” (P6)

##### Assistance provided by patients’ associations

Parents, in particular, explained that associations significantly helped them by providing reassurance and guidance (e.g., knowledge about albinism, safe spaces for experience-sharing…).

“We had a lot of questions. The lucky thing we had was that...we got answers. Thanks to the members of the association at that time. And then, when we needed it, we could always contact 2-3 points of contact that we had—in fact—with these families. They provided us with answers. They reassured us a lot.” (P2)

##### Support from friends

PWAs, in particular, emphasized the valuable part played by friends and classmates in providing academic support (e.g., note-sharing, help with reading the board) and emotional backing especially during periods of stigmatization.

“I got help from my friends: either we worked on the diagrams together, or they sent me theirs and I worked on my own—things like that. And yeah, I was very fortunate to have always been able to surround myself with understanding friends.” (PWA9)

### Parent-child dyadic functioning

In this section, we will elaborate on the key themes that arose when we asked participants to describe their relationship with their parent or child (Additional file 3: Table detailing thematic content analysis).

#### Unceasing parental support

Firstly, parents predominantly reported playing a large role in helping and supporting their child with albinism, notably through active engagement in the school environment. They exchanged with the school team to explain the unique challenges that their child with albinism faced and to determine the best possible accommodations. It was common for parents to emphasize the importance for their child to attend regular schooling.

Another crucial aspect was parental involvement in medical follow-ups. At this level, mothers were often the most involved (e.g., scheduling and accompanying to medical appointments).

Parents, in particular, reported their active involvement in assisting their child’s mobility and administrative procedures.

They also highlighted the importance of the emotional and psychological support they provided as a crucial resource.

“When I was little, it was my mother who would accompany me. Yeah, I don’t remember my father doing it. So it was my mother who would take me to the ophthalmologist.” (PWA8)

#### Parental concerns

One major concern that parents reported was being worried about their child’s safety. They also expressed worries about their child’s academic and professional future as well as concerns about potential peer rejection or discrimination.

Two noteworthy findings should be highlighted together: firstly, some parents expressed difficulty in connecting with the challenges experienced by their child with albinism. Secondly, a few PWAs explained that it was quite difficult for them to confide in their parents when they encountered difficulties.

“But for example, what I remember the most from primary school is when I had a period—actually—where I didn’t dare to talk to them about the problems at school and, in the end, I did. And actually, as soon as I did, I cracked: I had too much on my... it was tough.” (PWA2)

#### Parent-child disagreement and conflicts

Disagreements and conflicts are a common aspect of any parent-child relationship, but those with a child with albinism faced additional obstacles. For example, the impossibility for the child to practice some activities despite a strong interest (e.g., riding a motorbike).

While three parents reported few conflicts, one mother-son dyad had fluctuating relations that stabilized in late adolescence. Meanwhile, two mother-daughter dyads experienced significant strain, and the daughters often had to limit contact with their mothers in order to cope.

“The relationship has been tense at times. But... it’s very fluctuating depending on the moment. I feel that when it’s just the two of us, it goes very, very well, but if there are more—well—for example, if the whole family is gathered, I feel like it’s much more tense than usual.” (PWA7)

#### Shock upon diagnosis

Parents reported that both the discovery and diagnosis of albinism were experienced as a significant upheaval. Often overwhelmed with doubts and questions, sometimes, parents also felt guilty for transmitting the disorder to their child, thus adding to their emotional distress. One parent in particular had a hard time accepting his/her child’s disability. The only outlier pertains to the PWA whose diagnosis was made much later. In that case, the mother felt relieved upon receiving the news as it eliminated any lingering uncertainty regarding her child’s disability.

“We come home, we tell it to our husband who gets up saying, ‘Oh my God, what is this? Panic on board, ‘What have I done?’ That was his reaction, as a father who has made a child who is... well, you know. And he had to type ‘albinism’ on the internet and read terrifying things, so, well... we did our best.” (P1)

#### Prolix parents, reserved children

It was challenging for PWAs to provide detailed descriptions of their relationship with their interviewed parent, whereas the parents were more verbose when describing their relationship with their child. They used words like “complicity,” “trust,” “love,” and even “explosive” to describe their relationship.

Similarly, parents were forthcoming in describing the most notable personality traits of their child. Among the set of words used, two thematic trends seemed to emerge: on the one hand, traits that suggest that the child was organized, responsible, and detail-oriented (e.g., “mature,” “conscientious,” “perfectionist,” “demanding,” and “hard worker”), and on the other, words which refer to assertiveness and social confidence (e.g., “strong-willed,” “dynamic,” “leader,” and “extrovert”).

Furthermore, parents’ views of their child’s appearance could be multi-faceted. Some parents expressed relief that their child’s appearance did not conform to the stereotypical features commonly associated with albinism. In contrast, some parents embraced their child’s distinct features and considered fair hair and lighter colored eyes to be an attractive trait.

“He’s lucky to be rather... light brown-haired, he has blue eyes, and he has fair skin but not to the extent that people would question it, so in fact, I’ve never had a questioning look towards him.” (P3)

#### Two central but costly aims: autonomy and normality

**Fig. 3:**
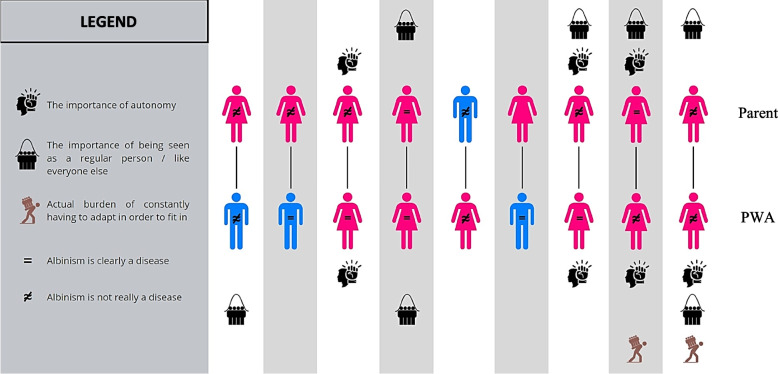
Dyadic functioning through transmission of parental leitmotiv and perceptions about albinism

Finally, one of the most interesting aspects highlighted in certain parents’ interviews was the presence of two key leitmotivs in their child-rearing practices, namely the “importance of autonomy” and “the importance of being seen as a normal person.” We also noted that, in those dyads, the children tended to adopt and integrate these recurrent themes into their own functioning (Fig. 3).

However, what is particularly noteworthy is that in the four PWAs who addressed the theme of “autonomy,” three expressed a burden of constantly having to adapt to be able to do things like everyone else (as detailed in the “main difficulties and obstacles” section). This can be a source of exhaustion that may have an impact on the overall quality of life of these individuals. In the discussion section, we will explore the factors that may explain the emergence of such a burden and what could potentially limit its impact.

“My parents made sure that I became independent quite quickly. And as a result, there are certain routines that maybe not everyone has, but it makes my life... kind of normal, you know.” (PWA9)

To conclude this results section, we provide a summary of the themes explored during the interviews. Table 2 encompasses all the themes addressed in our study and arranges them vertically based on their frequency (higher placement indicating more mentions). Horizontally, the table classifies the themes according to whether they were reported by PWAs or parents (i.e., themes reported by PWAs are positioned further to the left). The implications and further insights derived from this table will be elaborated upon in the “Discussion” section.

**Table 2:**
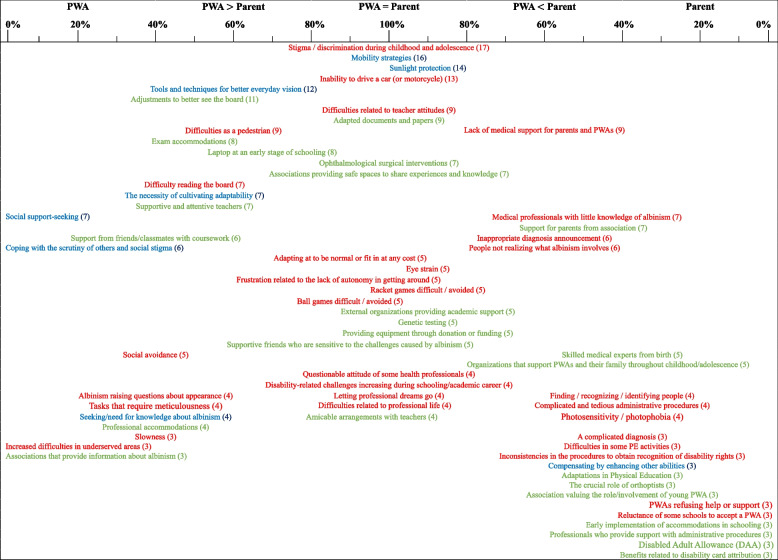
Overview of interview themes based on frequency and degree of concordance

The color of each theme is associated with a main section: difficulties and obstacles are in red; coping strategies are in blue; external sources of support are in green

The vertical position of a theme indicates its frequency among the interviewees: the higher the theme, the more frequently it was brought up. The number in parentheses shows the count of participants who referenced that theme

Below the labels of the first row, a scale is presented, representing the parent-child level of concordance on each theme. This scale is in percentages, 100% meaning that a theme was cited an equal number of times by both PWAs and parents and 0% that it was cited exclusively by either PWAs (left) or parents (right). In essence, the closer a theme is to the center of the table, the more it represents a shared viewpoint between PWAs and parents. Conversely, themes located at the extremities are more specific to either PWAs or parents

## Discussion

The nine parent-child dyads we interviewed have allowed us to identify several barriers and difficulties faced by PWAs as well as the resources they use to overcome these obstacles. We then gathered their own perceptions of their condition and the societal representations they perceive. Finally, we have described and analyzed the dyadic dynamics of parent-child relationships in the context of adjusting to disability.

Firstly, the themes we obtained align with the findings of a systematic literature review we conducted on the psychosocial implications of rare genetic skin diseases, including albinism [69]. We have found themes related to stigma and discrimination during childhood and adolescence, the importance of social support, genetic inheritance concerns, altered everyday functioning (e.g., mobility, skincare), difficulties with some healthcare providers, misconceptions and stereotypes in society, and a wide range of coping strategies.

As previously mentioned, Table 2 classifies vertically the themes from the most to the least frequent and, horizontally, indicates whether it is predominantly addressed by the PWAs or their parents.

Firstly, the theme related to peer social support was predominantly found among interviewed PWAs, and difficulties in interpersonal relationships tended to be primarily reported by them too. This outcome is unsurprising, given that parents have limited visibility into their children’s social lives, which consequently restricts their ability to identify elements that contribute to this thematic content. This finding aligns with numerous studies and literature that have demonstrated the importance of social relationships among friends and peers in disability adjustment and the well-being of affected individuals [70–72].

Secondly, interviewed parents tended to report difficulties they have encountered in their interactions with healthcare professionals, administrative structures related to disability support, and the educational system. To illustrate the latter point, it is noteworthy that only parents mentioned situations where certain schools displayed reluctance in accepting students with disabilities. Likewise, numerous studies examining the experiences of parents of children with a rare disease highlight these structural obstacles [73–75]. One of the main reasons the children never mentioned this obstacle might come from a protective intention from their parents. Indeed, parents may choose not to mention such situations with their children in order to protect them from such negative attitudes.

Concerning resources and facilitators, interviewed parents were more inclined to report the support they have received from healthcare professionals and specialized centers involved in albinism management as well as the reassurance and guidance provided by relevant associations during the first months of uncertainty following the diagnosis. This finding may be explained by the fact that parents, initially, are directly exposed to the shortcomings of healthcare and education professionals, whereas young PWAs have limited recollections of this period or may lack them entirely. Consequently, parents are more likely to express their satisfaction during interviews when they receive particularly strong support from medical professionals.

Finally, only the parents mentioned instances where their children categorically refused to use certain aids (e.g., classroom magnifiers). Parents, being highly attentive to ensuring their child has sufficient support may be more keenly aware of the accommodations that their children refuse to use. The reason for this refusal seems to be that the accommodation was either too cumbersome or too conspicuous, which could lead to potential stigma from their peers. Other researchers have already suggested this interpretation in studies involving young individuals with disabilities [76, 77].

Although numerous qualitative studies have explored the specific experiences of parents of children with disabilities [78–81], few have attempted to provide a dyadic qualitative approach to understanding disability experiences within the field of psychology. However, among the limited studies that have addressed this topic, some have indeed highlighted a differential focus between the narratives of parents and those of their affected child [82, 83].

The multitude of coping strategies developed by PWAs directly echoes the fact that they must constantly adapt to societal norms in order to be perceived as “normal.” Some have such an urgent need to conform that they go to great lengths to conceal their disability, which may exhaust them. Another recent study has obtained similar results in patients with visual impairments (VI) [84].

This daily burden experienced by PWAs has recently been conceptualized and can be measured using a validated scale [41]. The concept of “burden” emerged in scientific literature in the early 1980s as a mean to assess the perceived strain on caregivers who provide support to their elderly parents affected by dementia or neurodegenerative diseases [85]. The term was subsequently adopted by the World Health Organization (WHO) in the 1990s, as part of large-scale studies conducted across multiple countries to estimate the “Global Burden of Disease.” Now closely associated with the construct of quality of life, the concept of “burden” has been extensively explored and expanded to allow for its specific application to individuals directly affected by specific conditions, such as skin conditions [86] including infantile hemangioma [87], inherited ichthyosis [88], atopic dermatitis [89], and vitiligo [90].

Another notable finding of our study is the experience of stigmatizing and discriminatory situations during childhood and especially adolescence. Stigma, as we recall, encompasses a variety of discriminations that ultimately deny individuals or groups full social acceptance, reduce opportunities, and fuel social inequalities [91, 92]. Many studies have highlighted this phenomenon (and its various forms, such as self-stigma) in many chronic illnesses, whether they are visible, such as obesity [93, 94] or skin diseases [95, 96], or hidden, such as HIV [97]. Moreover, studies have shown how adolescence is a very sensitive period in terms of stigmatization [98, 99], a pivotal period where peers play a predominant role in the social development of the individual, largely influencing self-perception and identity construction [100, 101]. Hence, health-related stigma is a well-documented issue and a global barrier to health-seeking behaviors [102], engagement in care [103], and treatment adherence [97] across a range of health conditions [104, 105]. Given that many disabilities and chronic illnesses face these stigma-related issues, a group of researchers proposed a theoretical framework of stigma and discrimination applied to these conditions based on a corpus of studies examining leprosy, epilepsy, mental disorders, cancer, HIV, obesity, and being overweight [106]. This multilevel framework attempts to synthesize the general processes of stigma in a way that can be applied to various health issues. By incorporating contextual, personal, and experiential factors, this model appears to be promising for future research on albinism and, more broadly, rare diseases (see Additional file 4: Health stigma and discrimination framework).

Stigma can be accompanied by a number of misconceptions and false beliefs about albinism. Several participants refer to imagery imbued with mysticism, echoing the situation of PWAs in Africa. There, the issue takes on a different nature; albinism is much more visible in relation to the dominant skin color and numerous false beliefs hinder the experience of PWAs (e.g., superstitions regarding luck or misfortune, inhumanity of PWAs and death myth, etc.) [26, 107].

A chapter written by Kromberg and colleagues (2018) clearly highlighted these misconceptions. However, it has provided significant nuances on how these beliefs are distributed across the continent, especially in relation to the ethnic groups present in specific regions. In particular, the authors found that attitudes appear more positive in urban areas compared to rural ones, where myths are prevalent, superstitions remain strong, and traditional healers continue to have influence. The authors also attempt to interpret these myths and superstitions as a maladaptive response to managing individuals with disabilities within communities and society at large. The main concern here is that these myths have a dehumanizing effect on PWAs, who are targeted by extreme discrimination, including ritualistic crimes. Over the past 15 years, more than 700 attacks, including 241 murders, are reported to have occurred, primarily in Africa, although these estimates are likely underreported^4^. In France, few false beliefs have been reported (e.g., contagiousness, night blindness), but they remain ultimately anecdotal and appear to be in decline.

In western countries, researchers have rather primarily focused on VI when studying albinism [41, 108]. Additionally, the mobility problems that were identified in our interviews are also widely recognized in visual impairments such as glaucoma [109, 110], age-related macular degeneration (AMD) [108, 109] [111, 112], and diabetic retinopathy [113, 114]. Navigation errors and getting lost in unfamiliar environments can lead to recurrent stress and, ultimately, to anxiety that hinders social life [115, 116]. However, the use of electronic mobility devices has an extremely positive impact on the mobility of individuals with VI, as evidenced by feelings of increased safety, comfort, and reduced stress during travel, along with higher quality and increased frequency of travel [117]. Therefore, in terms of daily functioning, albinism is also comparable to ophthalmological conditions.

When considering all of the strategies implemented by people with disabilities to cope with the challenges they face, one concept that emerges is resilience. As conceptualized in health psychology, resilience is a complex and multifaceted construct that refers to an individual’s capacity to cope with and effectively adapt to stressful or difficult circumstances [118–120]. Research in recent years has suggested that resilience is a particularly relevant protective factor for people with physical disabilities [121–124] as well as for the parents of those directly affected [125, 126]. Resilience seems to be influenced by various factors, including internal or personal resources such as acquired skills (e.g., mindfulness and acceptance) and personality traits (e.g., optimism) as well as external resources such as environmental factors (e.g., social support) and cultural factors (e.g., traditions and cultural practices) [118, 119, 127–129].

While resilience was initially conceptualized as a protective factor in the context of acute trauma, recent research has suggested that it may also play a protective role in managing everyday difficulties related to chronic physical disabilities [123, 130]. However, even though research has demonstrated the beneficial effects of resilience in physical illnesses [131], our findings suggest that constantly adjusting to obstacles can be draining, and some persons in our sample reported exhausting their internal resources to the point where they received little external support. Does this highlight a key downside to resilience? The problem, in our view, is more related to the balance of the resources being mobilized. We will now offer a possible interpretation by integrating parental leitmotivs and disease representations into our reflection. As we have shown previously (Fig. 3), for the two interviewed PWAs who explicitly reported an actual burden, the theme of autonomy was present and seemingly learned from their parents. Conversely, when examining the interviews of the four PWAs’ who emphasized the importance of autonomy, the two others who did not report an associated burden saw albinism as a disorder. Thus, we hypothesize that while autonomy at all costs may enable individuals to adapt to their disability, it may also increase the burden associated with adaptability if they do not consider albinism to be a disorder. PWAs who prioritize autonomy would tend to adapt to daily difficulties despite the resources available, utilizing both internal and external resources to cope. So, what explains that, among these participants, some reported feeling an associated burden while the remaining participants did not? One possible interpretation is that there may be another variable to consider: whether or not albinism is considered a disorder. Thus, individuals who do not consider albinism to be a disorder or a source of disability may be more likely to only rely on their personal resources for adaptation, making little use of external resources and thus experiencing more exhaustion. To bolster this hypothesis, we will now delve into the narrative of PWA1. A closer examination of her interview revealed that her experience and views on albinism have changed over time. She reported having felt burdened during her high school years (page 14), which suggests that this was a challenging period for her to accept her condition. Yet, these years also corresponded to a period in her life when she did not consider her albinism to be a disorder, whereas today, her burden has subsided, and she even expresses belief in the importance of recognizing albinism as a disorder.

It is important to note that this interpretation of the results is just one possible hypothesis and would require verification through further research. Nonetheless, this interpretive framework resonates with many studies that have highlighted the strong link between individuals’ representations of their own illness and the type of coping strategies employed to deal with it [132, 133]. These studies exclusively rely on the Common-Sense Model of Illness Representations, which identifies five key dimensions of illness representations: identity, causes, consequences, controllability, and timeline [134]. While this model has led to the development of various questionnaires for its assessment [135–138], none of them explicitly inquire about the extent to which individuals perceive their condition as an illness or consider themselves as being ill or disabled. Therefore, it would be valuable to investigate this aspect in future quantitative research in order to explore the complex relationships between representations of albinism, the associated burden of albinism, resilience, and coping strategies.

Finally, Bronfenbrenner’s eco-systemic model served as a relevant framework in this study to explore and understand dyadic dynamics between the two partners. Additionally, it enabled us to highlight the pivotal role of the macrosystem in explaining the differential adjustment of PWAs individuals and their significant others (e.g., African context). We believe it is essential to take this model into account if we are to better understand the experiences of people with rare diseases, both in research and clinical settings.

### Limitations

In this study, several limitations are worth noting. Firstly, it is important to emphasize that our sample was heterogeneous, with various forms of albinism represented, and some not clearly reported nor included. Given this context, interpreting the results poses challenges due to the risk of overgeneralizing aspects that, in reality, may be more nuanced and pertain specifically to oculocutaneous albinism type 1 (OCA1) or type 2 (OCA2), ocular albinism (OA), or Hermansky-Pudlak syndrome (HPS) for example. A homogeneous group of participants all sharing the same type of albinism would have made the findings from this study more meaningful.

Secondly, we faced challenges in eliciting information from interviewed PWAs regarding their relationship with their parents. Often, their responses were vague and tended to generalize their feelings towards both parents when asked about their relationship with only the one interviewed. This also prompts us to consider the disadvantages of phone interviews compared to face-to-face interviews. Indeed, the former might result in a loss of information as non-verbal cues cannot be perceived. However, some authors suggest a more nuanced view, highlighting specific advantages and disadvantages for each method [139]. Moreover, in terms of interview content, other researchers have found no significant differences in the transcripts across the two investigative modalities [140].

Another limitation was recruitment bias. Our sample predominantly consisted of participants who were well-integrated into healthcare or community support systems. This meant that individuals who were more isolated or disengaged were underrepresented in our study. This can be attributed to the fact that the sample predominantly consisted of volunteers and self-selected individuals (incidentally, with all the parents being professionals) thereby further compromising the representativeness of our sample.

Furthermore, we observed a bias towards self-presentation among the families we interviewed. Some tended to present themselves as “perfect” families without any conflicts and often minimized or downplayed instances of stigmatizing behavior during childhood. This social desirability bias appeared to be particularly prominent when the parents described their children with very positive terms, emphasizing their resilience and strength in overcoming obstacles. This gave the impression that their children were depicted as champions, always ready to face any challenge. This aspect can raise questions because, even if this point of view is intended to be empowering, it can also perpetuate harmful stereotypes and reinforce the idea that people with disabilities must constantly strive to prove their value and abilities to others (i.e., modern ableism). It may be worthwhile to further explore and question this myth among families. By engaging in more candid and nuanced discussions, we may gain a more accurate understanding of the experiences and perspectives of individuals and families affected by disabilities.

### Perspectives

Firstly, we can note that one recurring issue in the field of rare diseases is the inadequate knowledge of physicians and the insufficient support they provide to patients and their families [33, 35, 141, 142]. While this problem has been documented in several studies, it must be highlighted that a large number of rare diseases have been identified (6000–8000), and each has its own unique and complex functioning. It is thus highly challenging for physicians to be experts in all of these diseases and provide optimal care for affected patients. So, in France, Rare Disease Reference Centers^5^ are dedicated to offering organized, consensus-based care. Some medical teams have recently developed guidelines to help practitioners in the diagnosis and care of albinism, such as a care plan that would begin as soon as the person is diagnosed^6^ [143].

To address these challenges, participants have proposed a number of avenues for improvement, including more comprehensive training for healthcare and education professionals, and the deployment of a secure diagnostic disclosure program, which would provide more empathy when the albinism diagnosis is announced to parents. Several additional suggestions were put forward by interviewed PWAs, such as improving healthcare coverage, including the possibility of reimbursement for specific expenses (e.g., sunscreens, prescription glasses and sunglasses), redesigning urban spaces with appropriate signage (e.g., auditory signals for crossing roads), reforming the visual impairment-related conditions for driving licenses, and revising the renewal timeline for disability rights. Only one of the parents suggested promoting the systematic use of low vision simulation glasses to raise awareness about VI.

Secondly, this paper underscores the importance of a systemic approach involving the main actors of the family sphere concerned and of biopsychosocial support for patients with genetic diseases. Treating these patients is a complex and time-consuming task that requires interdisciplinary collaboration between geneticists, specialists in affected organs, and general practitioners. French organizations specialized in sensory disabilities have already started to provide interdisciplinary support to young individuals affected by albinism and increasingly train their professionals in systemic approaches [144, 145]. A hospital team, together with a patient group (Genespoir), has also recently developed a Therapeutic Patient Education program [42]. This program offers long-term support that adapts to the evolving needs of the patient and their family. The program covers everyday life situations while also providing a platform for families to engage in dialogue, allowing them to discuss the obstacles they encounter and the solutions they have implemented.

Finally, it remains essential to continue studying the psychosocial experiences of PWAs, particularly in regions where very little research has been done so far (especially countries which predominantly focus on biomedical research). To address issues related to sample sizes and improve study designs, quantitative longitudinal lifespan studies could be established to gain a more detailed understanding of the developmental pathways of these conditions and identify factors that may influence them. Furthermore, action research or intervention-based studies could be conducted, such as prevention initiatives in schools and consultations or therapy sessions for parents, to implement effective interventions that directly benefit patients and their families.

## Conclusions

Albinism, like many health conditions, has significant impacts on various aspects of a person’s life and, in France can be comparable to other vision-related disorder. Albinism can also affect the family of the concerned person, especially the parents, who mobilize many of their resources to ensure their child grows up as normally as possible. Despite the challenges faced by PWAs, a vast majority of these difficulties are compensated for by the multitude of adaptation strategies they deploy, which can sometimes exhaust their personal resources and create a real burden associated with the disorder.

Therefore, it is crucial to consider albinism within a systemic and family dynamic framework to provide parents with a secure support structure and enable young PWAs to rely on various types of resources to avoid exhaustion. Further research should explore the longitudinal and developmental pathways of this condition and implement intervention-based studies to develop direct and beneficial interventions for the persons affected and their relatives.

### Supplementary Information

**Additional file 1.** NRIPH Attestation: this letter states that the project, led by Hugo Fournier and Bruno Quintard and conducted by the University of Bordeaux, is exempt from ethical review under French Public Health Code Article L1121-1.

**Additional file 2.** Semi-structured interview guides: A comprehensive guide outlining the questions and prompts employed in interviews with PWAs and the relative they’ve chosen for this study.

**Additional file 3.** Table detailing thematic content analysis: This table encompasses all main thematic domains, main themes, and sub-themes identified during the thematic content analysis. All main thematic domains and main themes are ordered in the same sequence as they appear in the text. The “Miscellaneous” section includes elements mentioned by only one participant.

**Additional file 4.** Health stigma and discrimination framework: This figure illustrates the model developed by Stangl et al. (2019), which seeks to synthesize the general mechanisms of stigma in a manner applicable to a diverse range of health-related issues.

## Data Availability

The datasets generated and/or analyzed during the current study are not publicly available due to the following reasons: Sharing qualitative data from interviews openly could compromise the confidentiality and anonymity of the participants, infringing upon their rights to data protection. The informed consent did not include provisions dissemination of all data from interviews in an open-access manner (even when anonymized), because it could be seen as a breach of the initial confidentiality agreement. Qualitative data (even when anonymized) may contain sensitive or personal information about the participants, potentially exposing them to psychological or social risks if made public. Nevertheless, in order to follow the ethical recommendations of qualitative research, the corresponding author may provide access to certain datasets upon reasonable request.

## Abbreviations

PWA: People/person with albinism
VI: Visual impairments

## Footnotes

1 The letters indicate status of the participant (PWA, person with albinism; P, parent), and the number refers to the dyad’s identifier.

2 Pedagogical integration units (PIUs) enable the collective inclusion in regular middle schools or high schools, of students who may struggle with the sometimes-substantial challenges of an individual integration.

3 SAAAS (in English, Service for Support in Autonomy and Education) is a French service that provides support and assistance to individuals with sensorial disabilities in order to promote their autonomy and facilitate their educational integration.

4 https://www.underthesamesun.com/content/resources

5 Rare Diseases Reference Centers (in French, Centre de Références des Maladies Rares) are specialized hospital teams that gather experts with proven expertise in a specific rare disease or a group of rare diseases.

6 Depending on the patient and the disease, the diagnosis can be more or less straightforward. Successfully diagnosing rare diseases is a very complicated task and is not devoid of errors or delays. In France, the Rare Disease Alliance conducted an investigation on diagnostic odyssey, revealing that a quarter of patients wait at least 4 years before receiving a definitive diagnosis [146].
