# Supplementary material for: The experience of albinism in France: a qualitative study on dyads of parents and their adult child with albinism
Source: BMC Med. 2024 Jan 29;22:40. doi: 10.1186/s12916-024-03251-z (PMC10823752; doi:10.1186/s12916-024-03251-z)
Supplement: Supplementary file 1 — Additional file 1. NRIPH Attestation: this letter states that the project, led by Hugo Fournier and Bruno Quintard and conducted by the University of Bordeaux, is exempt from ethical review under French Public Health Code Article L1121-1. [file 12916_2024_3251_MOESM1_ESM.pdf]

President

Bordeaux, 17th December 2021

To whom it may concern.

The University of Bordeaux is the host institution of a research project entitled «ALBIPSY: Albinism, Family & Quality of Life» whose scientific leaders are Mr. Hugo Fournier and Mr. Bruno Quintard. This research project is being conducted by the laboratory Bordeaux Population Health (Université de Bordeaux – Inserm U1219).

The provisions of the Article L1121-1 of the French Public Health Code are not applicable to this type of study and no ethical review of an "institutional review board" (IRB) is required under French legislation.

This research project is carried out in compliance with the Declaration of Helsinki, and according all good practices concerning information and consent of participants. This study was designed to be "privacy by design" and to comply with French data protection legislation.

For all legal purposes.

Manuel TUNON de LARA
President

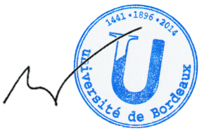

Adresse postale
Université de Bordeaux
351 cours de la Libération
33405 Talence cedex
www.u-bordeaux.fr
