# Supplementary material for: The experience of albinism in France: a qualitative study on dyads of parents and their adult child with albinism
Source: BMC Med. 2024 Jan 29;22:40. doi: 10.1186/s12916-024-03251-z (PMC10823752; doi:10.1186/s12916-024-03251-z)
Supplement: Supplementary file 2 — Additional file 2. Semi-structured interview guides: A comprehensive guide outlining the questions and prompts employed in interviews with PWAs and the relative they’ve chosen for this study. [file 12916_2024_3251_MOESM2_ESM.docx]

Semi-structured interview guide – PWA version

Can you give me three words that come to your mind when you hear the term “albinism”?

Can you share your story, how you grew up and lived with albinism?

- *Participants were prompted about specific periods of their lives when more information was required:* How was it at school? How was your transition into the working world?...

How do you manage your albinism in your daily life?

- What are your main challenges?
- What are your tips/strategies for adapting?

What can you tell me about the way you have been supported (medically, socially...)?

- Which healthcare professionals have been involved in your care?
- How is your relationship with these healthcare professionals?

How would you describe the relationship you have with your father/mother?

How does your daily life with your father/mother go?

- How do they support you?
- What are the things that can cause difficulties in your relationship?
- How do you handle tensions or conflicts in your relationship?
- How involved are they in managing your albinism?

Imagine that I am someone who knows little or nothing about albinism. How would you explain it to me in your own words?

Now, put yourself in the shoes of someone who knows little or nothing about albinism. Can you give me three words that come to your mind when you hear the term “albinism”?

In your opinion, what do people generally think about albinism in France?

How do interactions go with disability assistance institutions?

How do interactions go with patient associations?

If you had any suggestions or ideas for improving the quality of services provided by the organizations and institutions we have discussed during this interview, what would they be?

Semi-structured interview guide – Parent version

Can you give me three words that come to your mind when you hear the term “albinism”?

Can you share your son’s/daughter’s story, how did they grow up and live with albinism?

- *Participants were prompted about specific periods of their lives when more information was required:* How was it at school? How was their transition into the working world?...

How does your son/daughter manage their albinism in their daily life?

- What are their main challenges?
- What are their tips/strategies for adapting?

What can you tell me about the way your son/daughter has been supported (medically, socially...)?

- Which healthcare professionals have been involved in their care?
- How is their relationship with these healthcare professionals?

How would you describe the relationship you have with your son/daughter?

How does your daily life with your son/daughter go?

- How do you support them?
- What are the things that can cause difficulties in your relationship?
- How do you handle tensions or conflicts in your relationship?
- How involved are you in managing their albinism?

Imagine that I am someone who knows little or nothing about albinism. How would you explain it to me in your own words?

Now, put yourself in the shoes of someone who knows little or nothing about albinism. Can you give me three words that come to your mind when you hear the term “albinism”?

In your opinion, what do people generally think about albinism in France?

How does it go with disability assistance institutions?

How does it go with patient associations?

If you had any suggestions or ideas for improving the quality of services provided by the organizations and institutions we have discussed during this interview, what would they be?
