# Supplementary material for: The experience of albinism in France: a qualitative study on dyads of parents and their adult child with albinism
Source: BMC Med. 2024 Jan 29;22:40. doi: 10.1186/s12916-024-03251-z (PMC10823752; doi:10.1186/s12916-024-03251-z)
Supplement: Supplementary file 3 — Additional file 3. Table detailing thematic content analysis: This table encompasses all main thematic domains, main themes, and sub-themes identified during the thematic content analysis. All main thematic domains and main themes are ordered in the same sequence as they appear in the text. The “Miscellaneous” section includes elements mentioned by only one participant. [file 12916_2024_3251_MOESM3_ESM.docx]

| Main thematic domains | Main themes | Sub-themes | PWA | P | Relevant quotations (participant number) |
| --- | --- | --- | --- | --- | --- |
| Personal perceptions and societal representations of albinism | People's representations of albinism | Albinism being misunderstood or unknown to most people (e.g., limited awareness of VI) | 6 | 6 | *"But, in any case, it's not at all associated with visual problems, that's for sure. *stammers* Generally, people are a bit surprised: 'Oh really! Albino means they have visual problems?'" (PWA1)* |
|  |  | Misconceptions & Stereotypical beliefs (e.g., animal imagery; African mythology and folklore; stereotypical depictions in cinema and literature) | 5 | 6 | *"To me, it reminds me of a character in a movie... in Angels & Demons, there's a villain, a priest, and he's an albino. So we immediately recognize that... for me, that's what it is." (P4)* |
|  |  | Stereotypical “albino” appearance (e.g., albinism reduced to a physical stereotype: whiteness and red eyes) | 2 | 4 | *"The red eyes, white hair or pale skin. I think it's a bit of a stereotype as a result." (PWA3)*  *"But people don't realize that she's albino, because she's blonde, because she wears glasses, her nystagmus has stabilized really well: she still has it, but not too much." (P8)* |
|  | Definition | Visual Impairments | 8 | 7 | *"Actually, it's a depigmentation of the skin for albino people, ocular-cutaneous, with white hair, pale skin, and as a result, there are vision problems. There are two types of albinism, cutaneous and ocular. It causes us to have poor eyesight, light-colored eyes, and it's genetic. For instance, parents may carry a gene and pass it on to the embryo, which can lead to nystagmus, strabismus, and other vision problems. Low vision, photosensitivity also. There are also syndromes [...], there are several types, in fact, there are many types." (PWA5)* |
|  |  | Skin condition | 7 | 3 |  |
|  |  | High sun sensitivity | 5 | 3 |  |
|  |  | Genetic disease / condition | 4 | 3 |  |
|  |  | Lack or total absence of melanin | 3 | 3 |  |
|  |  | Transmission/inheritance | 3 | 1 |  |
|  |  | Hair system disorder | 3 | 1 |  |
|  | Whether albinism is seen as a disease/disorder or not | Albinism is not a disease or a disorder (e.g., terminology with strong connotations, implying viral involvement or lethality) | 4 | 6 | *"It's true that it's more of a 'particularity', yeah, I like the word 'particularity'." (P7)*  *"No, I wouldn't say it's a disease. Because a disease is a flu, a virus, that's what it is. It's not a disease." (P4)* |
|  |  | Albinism is a disease (e.g., albinism is, fundamentally, a genetic disease or disorder) | 5 | 2 | *"I think it's a disease because that's the definition of the word 'disease'. And I don't really see the point of not considering it as a disease." (PWA7)* |
|  | Albinism experience differing greatly depending on life context | Dangers associated with the African context (e.g., persecutions and massacres related to local superstitions; early abandonment of children with albinism; increased risks associated with sun exposure limited access to healthcare) | 1 | 3 | *"There is a social reality in Africa that is tragic, with children being abandoned, mutilated... So, in Europe, we have this fortunate circumstance, which is not insignificant." (PWA8)*  *"We have faced this because we have traveled quite a bit in Africa. Confronted - well - with children who were in situations - well - really with skin completely ravaged by the sun, who had no cap, there was no way." (P1)* |
|  |  | Completely different experiences depending on the era (e.g., less support and accommodations in the past) | 0 | 1 | *"At that time - now he is 18, I think the means are not the same now - but at that time, I felt alone in the world, in fact." (P3)* |
| Main difficulties and obstacles | Relationships with others | Stigma / discrimination during childhood and adolescence (e.g., stigmatization based on appearance; teasing, rejection, exclusion during younger years) | 9 | 8 | *"I think middle school was very difficult for her. In terms of social issues, she was rejected by her friends... and she took it very badly. [...] at that time, she was hoping that my husband would be transferred to another region because she was taking it very hard." (P6)*  *"In primary school, I remember there was this thing like, 'she looks like a granny because she has white hair'." (PWA9)* |
|  |  | People not realizing what albinism involves (e.g., surroundings may not understand the implications of VI and/or photosensitivity) | 2 | 4 | *"I suggested that it would be better to avoid the hottest hours and not go between noon and 2. And, well... I got the feeling that this person didn't understand that." (PWA9)*  *"As a sighted person, it's not easy to say: well, yes, he has low vision, but we don't really know what... what he can see." (P6)* |
|  |  | Social avoidance (e.g., past challenges in seeking assistance from others; avoids mentioning their albinism to others) | 4 | 1 | *"Well, it's true that I don't like it, I try to avoid the topic, I don't really like talking about it in front of others - I mean - I've always been uncomfortable with it, you could say." (PWA6)* |
|  |  | Albinism raising questions about appearance (e.g., nystagmus is surprising; Scandinavian origin?) | 3 | 1 | *"Um... for example, the trembling eyes. For the person you're speaking to, it can be a bit unsettling when they look at you." (PWA5)*  *"For me, people often ask if I'm Scandinavian." (PWA8)* |
|  |  | Finding / recognizing / identifying people (e.g., difficulty identifying people from afar) | 1 | 3 | *"Uh, I'm thinking of a particular difficulty in social situations: we often have trouble recognizing faces, especially from a distance." (PWA7)*  *"Oh, I remember when she was little and I used to pick her up from preschool, she wasn't sure if it was me outside..." (P8)* |
|  | Transport & mobility | Inability to drive a car or a motorcycle (e.g., driver's license ban) | 6 | 7 | *"Yep, it's also a problem when it comes to driving. I can't get my driver's license. So that's an issue too." (PWA3)* |
|  |  | Difficulties as a pedestrian (e.g., difficulty reading signage in public areas; easy to get lost due to VI; getting disoriented on public transportation) | 6 | 3 | *"I think when you don't see well, you get lost all the time: you don't know where you are, you don't know what the street is, you missed the bus stop, you took the wrong train... And actually, she would call me completely panicked, saying, 'I don't know where I am.'" (P1)*  *"In public transportation, sometimes what can be complicated is reading - for example - the platform number or the bus number, or things like that." (PWA4)* |
|  |  | Frustration related to the lack of autonomy to get around (e.g., frustration of being dependent on others for transportation) | 3 | 2 | *"Actually, when I was in the province, if someone who lived a bit far away said, 'I'll come pick you up,' I couldn't do it. And even today, I can't do it. It's a real... what I'm thinking is that I can't give it back. I could never say to someone, 'oh, next time I'll take you.' So it's a real struggle for me..." (PWA8)* |
|  |  | Increased difficulties in underserved areas (e.g., reliance on public transport almost impossible in rural/mountainous areas) | 3 | 0 | *"Well, for example, I live in the countryside, there is no public transportation." (PWA5)*  *"Where I live with my parents when I'm not in town for university, well it's true that there aren't many buses. It's once a day, let's say. So, it's not ideal." (PWA6)* |
|  | Difficulties related to health care providers | Lack of support for parents and PWAs (e.g., limited information on available aids/procedures; absence of explanation about albinism and its related symptoms) | 3 | 6 | *"Like, for example, my parents were not aware... they found out quite late about all the assistance we could get from the MDPH and that kind of thing. They found out when I was 13 or 14 years old." (PWA4)*  *"But things were not explained to me very clearly, like saying 'your child is visually impaired', things were not laid out like that. So, the medical support and guidance were really quite weak when it comes to that." (P7)* |
|  |  | Professionals with little awareness of albinism (e.g., doctors unaware about albinism's daily impact; inversion of the patient/expert roles) | 2 | 5 | *"But there are several doctors I've seen who had to be explained what albinism is." (PWA7)*  *"The ophthalmologist, um... he didn't know at all about albinism. So, he told us right from the interview: 'I don't know anything about it...'" (P3)* |
|  |  | Inappropriate diagnosis announcement (e.g., nonsense during diagnosis; lack of tact from doctors) | 2 | 4 | *"Actually, it's true that the doctors told my parents at that time that I wouldn't be able to pursue my studies, which of course turned out to be untrue." (PWA9)* |
|  |  | Questionable attitude of some health professionals (e.g., felt treated like a specimen/case study by doctors) | 2 | 2 | *"Because I remember this thing where they put a sort of helmet on her head with diodes, electrodes, and stuff. I thought, 'okay, that's fine...,' but... we don't have to do experiments every time she sees a doctor, even if it's for research..." (P8)* |
|  |  | A complicated diagnosis (e.g., albinism missed during newborn screening; disagreement among doctors) | 1 | 2 | *"They consulted several specialists who said that there was nothing wrong. And it was actually a neurologist - I don't remember where - who said, 'well, actually, it's albinism.' Not all the ophthalmologists agreed, and then they did a diagnosis and it was really albinism." (PWA5)* |
|  | Schooling | Difficulties related to teachers' attitudes (e.g., reluctant, inattentive or helpless teachers) | 5 | 4 | *"It was in middle school, an English teacher in 6th grade, she was also my main teacher, it was just horrible. Let's say she, um... she didn't want to do anything special, she didn't take anything into account, actually." (PWA9)* |
|  |  | Difficulty reading the board (e.g., challenges exacerbated in university due to the distance from the whiteboard/slides) | 5 | 2 | *"The teacher writes on the board and then we can't see anything, and so, in this case, I am a bit helpless because I just can't use it [...] anyway, math has always been a difficult thing for me - well - at least, especially during high school, it was already the subject where I felt that it was going to be complicated not to see the board." (PWA1)* |
|  |  | Disability-related challenges increasing during schooling/academic career (e.g., jump in difficulty when entering high school) | 2 | 2 | *"Actually - in middle school, academically, I was doing very well. But then, when I got to high school, I faced bigger difficulties where I really felt that not being able to see could handicap me." (PWA1)*  *"In middle school, it started to be a bit more complicated, because there were a lot more teachers, a lot more subjects." (P4)* |
|  |  | Difficulties in some Physical Education activities (e.g., racket/ball sports) | 1 | 2 | *"I didn't go through the process of requesting a certificate to prevent him from playing certain sports like badminton at the beginning of the school year. He was a bit confronted with that..." (P3)* |
|  |  | Reluctance of some schools to accept a PWA (e.g. director having concerns about potential impact on school statistics; advising specialized school) | 0 | 3 | *"I was thinking about a comment - a serious one - that a teacher made when she was in kindergarten. She came to me at the end of the year, she said, 'You know Mrs. M., you're going to have to move.' - 'Oh really?' - 'Oh yes, because she's going to have to go to a special school, you can't stay in a regular school.' So that was pretty abrupt, you see. You see, there you go, another comment that really got stuck in my craw. It's crazy, though, it's crazy." (P1)* |
|  | Activities that are difficult or avoided | Racket games (e.g., tennis, ping-pong, badminton) | 2 | 3 | *"The racket sports! Oh boy, that's really something! I can't hit the ball with my racket." (PWA3)* |
|  |  | Ball games (e.g., soccer, basketball) | 3 | 2 | *"Already, visual impairment and photosensitivity directly affect certain sports, such as ball sports." (PWA7)* |
|  |  | Tasks that require meticulousness (e.g., sewing, handiwork; graphic design) | 3 | 1 | *"I don't think I have very good fine motor skills in my hands, and on top of that, I'm not very careful... so if something requires being meticulous, like putting a product in my eye, that's not something I'm going to do, you know." (P8)* |
|  | Criticism of disability services agency (DSA) | Complicated and tedious administrative procedures (e.g., time-consuming; no support in the filing process; DSA almost impossible to contact; poor-quality web platform) | 1 | 3 | *""I tried to contact the MDPH: I called them but never managed to reach a service; they hung up on me, saying that it was complicated, and that I already had some help, which was already great... [...] I would say around 10 to 12 hours for putting together the application, and you can add an additional 4 hours for attempting to contact them, and then another 4 hours for writing letters." (PWA1)* |
|  |  | Inconsistencies in the procedures to obtain recognition of disability rights (e.g., biennial renewal whereas it's a permanent genetic condition) | 1 | 2 | *"For example, renewals: we know that albinism is a disease that is always present and may not improve, and it may even worsen over time. For instance, I have a RQTH that I will need to renew in 2022. However, my disability will always be there throughout my life." (PWA5)* |
|  | Life aspirations and choices | Letting professional dreams go (e.g., VI as a barrier to certain careers: military) | 2 | 2 | *"I wanted to join the army, that was my life goal, you could say. I really wanted to, but I can't. [...] So, I wasn't feeling very well, yeah... It was a bit of a darker period." (PWA3)* |
|  |  | Compelled to live in an urban zone (e.g., difficulty commuting to work in rural areas) | 0 | 2 | *"They know they won't be able to settle in the countryside, it's not possible." (P4)* |
|  | Adapting to be normal or fit in at any cost  (e.g., burden from constantly adapting without speaking up about difficulties; refusing some aids to be perceived as normal; paradoxical desire: wanting to be normal / having an abnormality) | | 3 | 2 | *"Is it always a good thing for me to constantly be in a state of adaptability and not always talk about the underlying difficulties of my albinism? Because I used to have a very positive outlook where I kept all the difficult things to myself and didn't talk about them. [...] The real challenge was the way I looked at myself, I think. How capable I believe I am; to what extent I position myself as 'You can do anything, but at the same time, you can't do things like everyone else.' I think that's really it... yeah." (PWA1)* |
|  | Eye strain  (e.g., fatigue/headaches due to the effort required to compensate for VI; headaches exacerbated by nystagmus; fatigue exacerbated by white backgrounds: screen, papers) | | 2 | 3 | *"It was a lot of fatigue on top of everything else because such visual efforts were causing fatigue, you see? […] You have to make a constant effort to see, to perceive, to apprehend all that." (P8)* |
|  | Difficulties related to professional life  (e.g., VI exacerbating challenges in finding a job: mobility limitations; doubts about being recruited based on skills versus disability status; precarious employment contracts) | | 2 | 2 | *"For example, we can't drive, and all the offers for archivists that require a driver's license, I don't apply for them because I know very well that I couldn't have it." (PWA4)*  *"She works, unfortunately, on fixed-term contracts because that's all she can find, but she deals with it. The contracts follow one another, and she is not without work." (P4)* |
|  | Photosensitivity / photophobia  (e.g., increased brightness worsening visual abilities; pain due to sudden changes in brightness; more or less livable places depending on the time of day) | | 1 | 3 | *"So, it's true that the daylight can be a bit complicated for me. stumbles And then, in addition, we don't have curtains - well, it's a bit complicated - but we don't always have curtains at home, so that's a bit annoying: there are times of the day when I don't feel well in certain rooms." (PWA8)* |
|  | Slowness (e.g., need for more time to do some tasks) | | 2 | 1 | *"It's really the pace, the slowness. I do things quite slowly." (PWA9)* |
|  | Refusing help / support (e.g., rejecting sun protection measures; declining a school support assistant) | | 0 | 3 |  |
| Main resources and facilitators | Development of a variety of adaptation/coping strategies | Mobility strategies (e.g., public transportation; personal transportation alternatives: bike; support from family/friends for car transportation; asking passersby for help; smartphone for wayfinding; anticipating extra time for trips) | 8 | 8 | *"I also ride a bike, it serves as my vehicle instead of a car." (PWA2)*  *"We do our best when she has an outing: we take her or friends come to pick her up." (P6)*  *"Now, I ask for help, which I didn't do before. So, in the street, if I don't see a street, I say, 'hello, excuse me, can you read me the name of the street?'" (PWA8)*  *"I use my phone a lot, especially for transportation, navigating, etc." (PWA7)* |
|  |  | Sunlight protection (e.g., sunglasses/tinted glasses; highly protective sunscreens; minimize sun exposure; clothing strategies: hats/caps, long-sleeved shirts) | 6 | 8 | *"Well, there was always sunscreen available... we always had a sun hat and sunglasses. And I know they were always looking for a spot with shade for us, to avoid being too exposed. But I remember when we went to the beach, we would avoid going during peak sun hours around noon. Because that's when the sun is the strongest, the most intense, so..." (PWA4)* |
|  |  | Tools and techniques for better everyday vision (e.g., low vision glasses; smartphone camera zoom function; getting very close to screens or other people's faces; portable monocular/binoculars) | 8 | 4 | *“I take a picture of the panel with my phone, and on my phone, I can zoom in on the picture and read the platform number, for example." (PWA4)*  *"I only used a magnifying glass, and I had plenty of them, various magnifications, with lights, without lights. So that's what I used on a daily basis..." (PWA8)*  *"I take pictures and zoom in on my phone. Same for signs when we walk, for example, direction signs. I take a picture and then I look at it." (PWA5)* |
|  |  | The necessity of cultivating adaptability (e.g., adaptability facilitates overcoming challenges and achieving the most difficult tasks) | 5 | 2 | *"This forced adaptability has a positive side in that I have always adapted and I know how to adapt, and it's actually complicated to know how to adapt to everything because we don't have limits to adaptation, we accept everything, we end up being capable of accepting and saying: 'Ok, I need to adapt? Well, I'll adapt'." (PWA8)* |
|  |  | Social support-seeking (e.g., asking for help to read/see something, or to learn how to use certain tools) | 7 | 0 | *"Today, I have this approach: I go to people and tell them, 'Well, here is my issue, and to make things go smoothly, I need this or that. And if I encounter a problem, I will let you know so we can resolve it and make things work out for everyone.'" (PWA4)* |
|  |  | Coping with the scrutiny of others and social stigma (e.g., explaining albinism to others to avoid misunderstandings/comments; support from friends to cope with stigma; humor and self-deprecation to diffuse tension) | 6 | 0 | *"I used to laugh a lot at myself, which made the topic much lighter and much less complicated - in fact - for the people around me, I think. Even for my friends, and that's what they often told me later: "In fact, we felt pretty comfortable with your disability because you assumed it so much and laughed at yourself so much that - in fact - we quickly got over it." [...] I made sure that others didn't have a pitiful look at me and had a rather positive and almost admiring look, actually." (PWA1)* |
|  |  | Seeking/need for information about albinism (e.g., online resources about albinism; knowledge acquired through the albinism association) | 3 | 1 | *"The fact of attending conferences interested me a lot because... it helps to understand better and, well, I really like to inform myself, to know more, I'm quite curious about these kinds of things." (PWA2)*  *"Well, now I would say that it's better because, well, first, the Internet helps a lot. And the advantage is that... well, there is a little more information available." (P2)* |
|  |  | Compensating by enhancing other abilities (e.g., hearing; memory; computer skills) | 1 | 2 | *"So, he memorized what the teacher said in his head - and he didn't have to repeat it much, because since it was written on the board, the teacher wouldn't repeat it, so he memorized it, and then copied it." (P7)* |
|  |  | Miscellaneous (e.g., concealing albinism with makeup or hair dye; development of a fighting spirit; strategies to identify people: clothing color) | 2 | 2 | *"Since nobody really knew, I wasn't stigmatized... you know, with my monocular I was too embarrassed. I wouldn't write on the typewriter, it was all very hidden." (PWA8)*  *"So, he had one or two friends he would spot with their coat, the color of the coat." (P3)* |
|  | School accommodations & adjustments | Adjustments to better see the board (e.g., seating in the front row or closer; video magnifier; allowed to move around to better see what's written on the board) | 8 | 3 | *"We were really very close to the board, we were not even a meter away or something like that." (PWA4)*  *"So I had a kind of big computer that allowed me to see the board. Generally, I didn't like it." (PWA1)*  *"He also had the right to move around the class and get closer to the board." (P7)* |
|  |  | Adapted documents and papers (e.g., enlarged documents and papers; better font choice; adapted paper background: paper color, grid) | 5 | 4 | *"Meaning that several students benefited from it, and we didn't all necessarily have the same needs. I know that I like Arial 16 - for example - and others preferred 18 or 24 or Comic Sans MS - anyway - everyone has their own adaptation. And it's true that it was quite... that it was quite good to have that." (PWA9)* |
|  |  | Exam accommodations (e.g., extra time; separate exam room; exam administered on a computer) | 6 | 2 | *"In terms of studies, I just had an additional time for the exams of the baccalaureate and the diploma." (PWA7)*  *"When I passed exams again, I composed on computers.” (PWA8)* |
|  |  | Laptop (at an early age) | 5 | 3 | *"I had a laptop since 5th grade... so with that laptop, I would take notes for all the explanations, I tried to take good notes in class, that was easy." (PWA9)* |
|  |  | Supportive and attentive teachers (e.g., transcribing certain course content; giving advices to help in everyday life) | 5 | 2 | *"There were some teachers who were understanding and would come write certain things for me." (PWA4)*  *"I had a math teacher who was visually impaired himself, and he really helped me. He suggested some software for magnification and text-to-speech to help me read better, like ZoomText." (PWA9)* |
|  |  | Outside structures providing support (e.g., specialized educators offering complementary activities; the role of these structures in informing schools about albinism) | 2 | 3 | *"At the time, there were two women who followed the children at school, [...] who attended the classes at the same time as her and made her do small exercises of... you know, drawings, things like that.” (P8)*  *"Well, the fact that outside experts would come to the school to explain what my daughter could see, what type of pencil to use so she could perceive the board better or what to do..." (P6)* |
|  |  | Typewriter (at an early age) | 2 | 2 | *"Plus, in 6th grade, I arrived with my little typewriter on which I had learned to type... I learned to type when I was 7." (PWA9)* |
|  |  | Adapted desk (e.g., adapted desk design with incline for posture and expanded surface for large books) | 2 | 2 | *"They made a desk for her, which was slanted so that she could... - oh yes! - it needed to be big, for having big notebooks. It needed to be elevated and slanted to avoid straining her neck. To prevent bad posture." (P9)* |
|  |  | Amicable arrangements with teachers (e.g., explaining needs to teachers to find adjustments in class; asking their help to complete notes) | 2 | 2 | *"I was always able to let the teachers know and intervene when needed, sometimes more discreetly, saying 'watch out, this was a bit difficult for me'. [...] I would take notes and check with the teacher at the end of the class, if necessary." (PWA1)* |
|  |  | Adaptations in Physical Education (e.g., exemption from evaluation; colorful oversized balls/birdies) | 1 | 2 | *"And before, we played badminton. But often the teachers still understand. So either they give me a bigger ball, or I become the referee. So I'm not graded." (PWA3)* |
|  |  | Early implementation of accommodations in schooling | 0 | 3 | *"During her time in preschool, there wasn't much in the way of accommodations, but as soon as she started elementary school, there were supports put in place." (PWA6)* |
|  |  | Miscellaneous (e.g., Teaching Assistant (TA); Specialized Units for Inclusive Education (SUIE); procedure to avoid sudden light changes; permission to rest eyes in class) | 1 | 3 | *"When they used the projector, they had to close the shutters and then turn on the projector. And they had to do it in a certain order so that he wouldn't be dazzled and it wouldn't hurt his eyes. There couldn't be a too abrupt transition." (P7)*  *"We were lucky enough to be able to go to a high school with a SUIE, a specialized unit for the visually impaired. So there was a teacher who managed all the classes for them." (P9)* |
|  | Healthcare and social support services | Ophthalmological surgical interventions (e.g., reducing nystagmus; correcting strabismus) | 3 | 4 | *"I believe it was the doctor who had told us that we needed to undergo surgery to improve our vision and reduce the nystagmus." (PWA4)* |
|  |  | Genetic testing (e.g., knowing the probability of having a child with albinism) | 2 | 3 | *"We consulted geneticists who told us that if we had other children, there was a one in four chance that they would have the albinism condition, that's it." (P8)* |
|  |  | Skilled medical experts from birth (e.g., prompt diagnosis shortly after birth; directed straight to the appropriate healthcare professionals; encouraging parents to raise the PWA “normally”) | 1 | 4 | *"Right away, we were told that our child would have visual impairment, but not blindness, and they could follow a normal school pathway with a bit of equipment and some human assistance, you know. [...] And that made it easier for us to approach her first five years." (P2)* |
|  |  | Structures that support PWAs and their family throughout childhood/ adolescence (e.g., parental support and guidance; psychological support; transdisciplinary support) | 1 | 4 | *"It's true that we had good support each time from SAAAS, which allowed her to have a normal education and for there to be facilitator who came to help her and explain what visual impairment was to the school. Because it's true that it's a disease that isn't well-known, and that teachers aren't familiar with. That's why these visual impairment centers are really well-made." (P6)* |
|  |  | Professionals who provide support with administrative procedures | 0 | 3 | *"For example, we filled out the documents for the baccalaureate, for the accommodations for the diploma. There's no need to make an appointment, they filled it out for us between two appointments, it's quite helpful." (P3)* |
|  |  | The crucial role of orthoptists (e.g., visual rehabilitation) | 1 | 2 | *"Until about midway through high school, she also had an orthoptist who was following her for eye rehabilitation. But now she doesn't need it anymore, you know." (P6)* |
|  |  | Miscellaneous (e.g., regular check-ups for the evolution of moles with dermatologists; doctors advising parents to get in touch with a patient association; medical professionals easing concerns about the future of PWAs) | 1 | 3 | *"And this doctor told her: 'You know, I have patients who are older than you, and you know,' he said to me, 'they're about to take their exams, don't worry, you'll see, you'll do it like everyone else'. And I remember all of that very precisely, you know." (P8)*  *"All the skin follow-up to see - because she has moles that appear, so we also monitor that to see if there are any changes, yeah." (P6)* |
|  | Support systems for disabilities | Providing equipment through donation/funding (e.g., funding provided by a specific institution; financial support for educational equipment) | 2 | 3 | *"She had assistance because...she quickly started using computers, so they quickly loaned her computers." (P6)*  *"During my Master's degree in Literature, I applied for funding from Agefiph to obtain a laptop." (PWA8)* |
|  |  | Disabled Adult Allowance (DAA) | 0 | 3 | *"She still receives a little bit of DAA, but not much since she is working, so yeah." (P4)* |
|  |  | Benefits related to disability card attribution (e.g., priority for front row seats at shows/cinemas; preferential/free access to museums and parks; guidance from airport staff) | 0 | 3 | *"While she has a disability card, which allows her... well, as soon as we go to a show or to the cinema, she goes in front: it allows her to have seats in the front." (P6)* |
|  |  | Recognition of Handicapped Worker Status (RHWS) (e.g., benefits related to the Obligation to Employ Disabled Workers) | 2 | 0 | *"They told me outright that my disability recognition was good for their numbers. So, when I went to meet with the head of the department where I work, he told me clearly." (PWA4)* |
|  |  | Miscellaneous (e.g., programs/events that promote professional integration for people with disabilities; funding support for ocular surgery) | 1 | 1 | *"I recently participated in something called the Duo Day [...]. So, basically, you spend a day in a duo with a person in the company, and the idea is to create an exchange - between the person with a disability and the company - to reflect on what are the interesting accommodations for disabled people in companies, and for disabled people to learn about the business environment, to get informed, and so on." (PWA1)* |
|  | Support from associations | Support for parents (e.g., providing reassurance to new parents; answering parents' questions; guiding parents towards competent centers/physicians; distance support as the main form of assistance; help with disability-related administrative procedures) | 2 | 5 | *"We had a lot of questions. The lucky thing we had was that...we got answers. Thanks to the members of the association at the time. And then, when we needed to, we could always contact 2-3 points of contact that we had - in fact - with these families. They provided us with answers. They reassured us a lot." (P2)*  *"We quickly said to ourselves: 'Ok! Wonderful, in the end'. This baby is going to become a little girl, a young girl, a young adult, and there you go, she will evolve like all these people, and it was very structuring and very important for us." (P1)* |
|  |  | Providing safe spaces to share experiences and knowledge (e.g., annual friendly meeting event; organization of occasional meetings between some families; Q&A online forum) | 4 | 3 | *"Because every year, being part of an association for two days, where suddenly, you're not a different person among others, but rather, you're just like everyone else. And it's actually the non-albino people who end up feeling different from everyone else. I think that for an 8-year-old girl, it's extremely important. It's crucial that for just two days out of the year, everyone understands us, everyone understands each other. The labels and names are written in a font size 18, which is really nice." (PWA1)* |
|  |  | Valuing the role/ involvement of young PWA (e.g., support groups for young people to discuss common issues) | 1 | 2 | *"Now it's even meetings between young people their own age, where they share their own answers and questions, compare their experiences and involve younger ones who may have the same question in the future." (P2)* |
|  |  | Associations that share knowledge about albinism (e.g., annual meetings featuring scientific conferences) | 3 | 0 | *"Attending conferences really interests me because it helps me to better understand, and I really enjoy learning, being informed and being curious about these kinds of things." (PWA2)* |
|  | Support from friends | Support from friends/classmates with coursework (e.g., sharing their notes; helping to read/see what's written on the board) | 5 | 1 | *"I got help from my friends: either we worked on the diagrams together, or they sent me theirs and I worked on my own - things like that. And yeah, I was very fortunate to have always been able to surround myself with understanding friends." (PWA9)* |
|  |  | Supportive friends who are sensitive to the challenges caused by albinism (e.g., emotional support from protective friends; making compromises with friends about certain activities; sharing tips and tricks with other VI friends) | 3 | 2 | *" My sister studied in a school for the visually impaired, which I didn't. And we were talking about little tricks that each of us had - once they came to visit me, and we talked about little tricks we had to take public transport." (PWA4)*  *"I'm thinking of one of her very old and close friends, she was in a very small class with her, and she remains her very close friend who was - I think - maybe overprotective with her between the ages of 4 and 15, you see." (P1)* |
|  | Essential passions or hobbies in the life of the PWA | Sporting passion (e.g., self-transcendence; coach who facilitates social interaction; sports communities inclusive towards disabilities; sports providing an outlet) | 2 | 2 | *"Well, from a sports perspective - because I think it's really an area that pushes us to surpass ourselves a lot, and puts us in situations where - in fact - our disability will be more of a positive than a negative point." (PWA1)*  *"When she joined the horse riding group, the instructor wanted to see a little bit of what my daughter could see, to understand what her visual impairment was like." (P6)* |
|  |  | Artistic passion (e.g., music is a space where disability can be turned into an asset) | 1 | 1 | *"This is really an area where my poor eyesight has led me to think about other ways of playing music, and in fact, it has been a huge positive point because it has given me an ear, it has given me a sensitivity." (PWA1)* |
|  | Professional accommodations  (e.g., personalized working methods with digital tools; adjusted schedules) | | 3 | 1 | *"I had the perfect situation because I had chosen my schedule - well, I didn't work on weekends, I had regular hours, like 10am-4 or 5pm, which was very suitable, in fact." (PWA8)* |
|  | Improved media coverage/distribution of information about albinism  (e.g., increased visibility due to social media; albinism is becoming a beauty standard) | | 1 | 1 | *"There are quite a few photographers who have used albino models, so I think it's a perception that has been around for a while, but I believe it will evolve with time. Especially with social media and communication being made about it." (PWA1)* |
| Dyadic functioning (or parent-child functioning) | Resources and support from parents | Involvement in schooling (e.g., communication with school team to find the best accommodations; importance of following standard coursework; involvement in managing course resources; individualized lessons; preferring private schools; parents reducing work hours for child's educational support) | 4 | 9 | *"There were teachers who were disability coordinators - so they were more trained in everything related to dyslexia - so they would say to me: "Oh well, we'll put lots of colors and everything!" And I said: "Oh no no no! You just put black and white, that's it". *laughs* "He's not dyslexic, he's visually impaired." So from there, there was real support, everything was done well." (P7)*  *"At first, we helped them a lot, especially for coursework, and then I went part-time." (P4)*  *"We've always favored private schools so that it's... more personalized so that classes - well - have fewer students or things like that." (P6)* |
|  |  | Involvement in medical monitoring (e.g., often mothers scheduling, managing, and bringing their child medical appointments; encouraging routine dermatologist visits) | 5 | 6 | *"When I was little, it was my mother who accompanied me. Yeah, I don't remember my father doing it. So, it was my mother who took me to the ophthalmologist." (PWA8)*  *"Today, I told her: "It wouldn't be a bad idea for you to see a dermatologist anyway" - "Oh no, it's pissing me off!..."” (P1)* |
|  |  | Support with moving around (e.g., transporting their child by car; support with planning transportation in advance; accompanying them on site or route reconnaissance; teaching their child to find their way if they get lost during travel) | 2 | 4 | *"I just said it was about transportation. We had to take them, bring them back when there were no buses, no means of transportation... But it was a choice we made." (P2)*  *"So, I always say: I taught her how to get lost, that's it: "Okay, I don't know where I am, I'm going to handle it calmly, [...]". So I taught her not to panic when she get lost, and she handled it with a little more calm..." (P1)*  *"If he goes to an unfamiliar city, he needs my presence. And even to go to ***, which is 30/40 kilometers away, I accompany him." (P5)* |
|  |  | Involvement in administrative procedures (e.g., fighting to defend the rights of their child; young people not inclined to deal with administrative paperwork) | 1 | 4 | *"I find that you often have to fight to assert your child's rights, actually. Well, I see it as a parent." (P6)*  *"But, like many young people today, the paperwork for health insurance, paperwork for things like that... it's the kind of thing that goes over her head. Well, I also help her with that." (P4)* |
|  |  | Emotional and psychological support (e.g., supporting their child through peer rejection; comforting them when difficulties cause a dip in morale) | 1 | 4 | *"When he came home in tears saying, "I was deprived of recess, I couldn't dissect a frog." But I told him, "Wait, it's not possible, have you tried to dissect a frog?" So we did everything behind the scenes: I helped him calm down, made him realize that it was really not possible, and all that..." (P7)* |
|  |  | Listening and paying close attention to their child's disability-related needs (e.g., anticipating needs and finding compromises; going to the beach outside of peak sun hours) | 2 | 2 | *"We simply adapt, you know: we don't go to the beach at 3pm in the afternoon, but we go at 5pm for example. Which is just as nice by the way." (P2)*  *"I found that they were always looking for little tricks to make us as comfortable as possible. Or when we said: "we're dazzled", or "we can't see". - "Well, wait, let's try to move elsewhere" and that's it." (PWA4)* |
|  | Challenges related to parenting | Concerns/worries regarding their child (e.g., concern for their child's safety while biking or moving around the city on their own; worries about their child's future; sadness about their child facing peer rejection) | 1 | 5 | *"I think there's something ingrained in me that makes me very anxious when I know she's crossing the street." (P1)*  *"But it's just that, with regards to parents, the worry that parents have when they see their children growing up. How will they project themselves? ... you know, this transition to the adult world, where we wonder, what will happen next?" (P3)* |
|  |  | Difficulty connecting with their child's difficulties (e.g., struggling to understand their child's visual difficulties; tendency to forget the challenges of albinism) | 1 | 4 | *"Because we didn't fully understand how difficult it was for him to see contrasts properly." (P7)*  *"And even in the family... Sometimes we forget about her visual impairment. Because she does so many things like a so-called 'normal person' [...] and sometimes we forget, well, that she has this visual problem." (P6)* |
|  |  | Differences in treatment can create tension when siblings do not share the condition of albinism (e.g., siblings without albinism may feel overshadowed when albinism takes all the attention; feeling excluded when all other siblings share albinism) | 2 | 2 | *"But regarding his sister, I would say that there is... How to explain? She feels like I have neglected her compared to her brother, but no, it's... I gave him more attention, [...] but... Yes, she carries this little reproach within the family." (P5)*  *"Well, with my older sister, I think she's the person who didn't handle this disability very well. I think she had a feeling of - well - since our parents spent a little more time with us to adapt to our disability. I think she may have felt abandoned a bit by our parents..." (PWA4)* |
|  |  | Opening up to parents can be a challenge (e.g., difficulties in confiding school-related problems to their parents) | 3 | 0 | *"But for example, what I remember the most from primary school is when I had a period - actually - where I didn't dare to talk to them about the problems at school and, in the end, I did. And actually, as soon as I did, I cracked; I had too much on my... it was tough." (PWA2)* |
|  |  | Miscellaneous (e.g., feeling unsupported by parents during difficult times; early parentification of the child with albinism) | 2 | 1 | *"So there were times when I didn't feel entirely protected and a bit alone in the world because I was told so much: 'Go on, be independent, be independent', that sometimes, I felt: 'OK, but can't you be here? Because I don't want to be independent right now, I need you by my side'..." (PWA1)* |
|  | Parent-child friction/conflict | Conflict triggers (e.g., limited academic involvement; using a motorbike for transportation; household tasks; endless criticisms and reproaches) | 3 | 5 | *"Sometimes it can also annoy me when we talk about motorcycles or something. Since I was little, I've wanted to ride a motorcycle, but I can't because of my disability." (PWA3)*  *"If it's just going to lead to hearing 5000 comments about what I've said or done, and then it becomes a topic of criticism... I don't want this." (PWA8)* |
|  |  | Conflict management (e.g., limiting contact/keeping a safe distance; avoiding topics that may lead to conflict; (explosive) arguments followed by reconciliation; peaceful resolution through dialogue) | 2 | 4 | *"Well, I think every person needs to take a step back from their parents at some point in their life." (PWA9)*  *"So it's not our thing to stay in a situation of unspoken issues... no, we need to address the problem, there are always solutions." (P4)*  *"No, I don't want any conflicts. I try to avoid conflict as much as possible. I don't want to get into a conflict with her." (PWA8)* |
|  |  | Almost no conflict (e.g., a few minor disagreements; impossible to be in conflict with their child) | 0 | 3 | *"No, no, we've never had any conflicts, actually. We've never had major disagreements about things. Well, from time to time, it's a bit normal, but not more than that. [...] I'm lucky, you know, I haven't had any conflicts with my daughters. The few disagreements we have, sometimes they're really minor." (P2)* |
|  | View of oneself/their child | Attitude towards appearance (e.g., fortunate their child doesn't exhibit typical albinism traits, minimizing impact on their self-image; loving their child's appearance; not completely albino as their appearance is less pronounced) | 0 | 6 | *"He's lucky to be rather... light brown-haired, he has blue eyes, and he has fair skin but not to the extent that people would question it, so in fact, I've never had a questioning look towards him." (P3)*  *"But she was so beautiful, you know? There's this aspect of 'wow, she's not like the others'. But I also tell her, 'you know, a very pretty girl is not like the others either, and everyone turns their heads towards her too'." (P2)* |
|  |  | Personality traits (e.g., mature, conscientious/perfectionist, strong-willed, dynamic, leader, demanding, extraverted, anxious, helpful, hard worker, shy, reckless...) | 0 | 6 | *"She's extremely demanding, she puts pressure on herself, she always wants to be at the top, she can't stand being in mediocrity, there's a lot of demand and work; I've spent my life telling her: "let go, it's okay!"" (P1)* |
|  |  | Negative self-image in adolescence (e.g., very low self-esteem, believing they will never experience any intimate relationships) | 2 | 0 | *"Especially on self-perception, sexuality, etc. If you had asked me the question at 15-20 years old, I would have given you a completely negative answer. Because, yes, at 20 years old, I thought I was going to die a virgin, and that no one would love me and it would be horrible." (PWA8)* |
|  | Transmission of parental leitmotiv | Importance of autonomy (e.g., parents aspiring for early autonomy and independence in their child's life; striving to rely as little as possible on others) | 4 | 3 | *"My parents made sure that I became independent quite quickly. And as a result, there are certain routines that maybe not everyone has, but it makes my life... kind of normal, you know." (PWA9)*  *"What we wanted to teach our daughters is how to fend for themselves in life." (P4)* |
|  |  | Importance of being seen as a normal person/like everyone else (e.g., parents' wish for their child to be considered like everyone else; PWAs sees themselves/wishes to be seen as a normal person) | 3 | 4 | *"We've always pushed our three children, and she's always followed along like everyone else, you know." (P6)*  *"I am fully integrated, you see. I am blond, I am not really affected by albinism. I am blond with glasses, like many people. I am just like everyone else, really. I am incognito, you know." (PWA8)* |
|  | Upheaval upon discovery and diagnosis of albinism  (e.g., disturbing diagnosis which can cause worries/panic; overwhelmed by doubts and questions after the announcement; feeling guilty for transmitting the disease to their child) | | 2 | 6 | *"We were very worried. A lot of questions, a lot of worries, and of course, guilt, for several years, I would say, because it's not... well... it takes a little time, anyway." (P2)*  *"We come home, we tell it to our husband who gets up saying, 'Oh my God, what is this? Panic on board, 'What have I done?' That was his reaction, as a father who has made a child who is... well, you know. And he had to type 'albinism' on the internet and read terrifying things, so, well... we did our best." (P1)* |
|  | Description of their relationship with their child  (e.g., complicity, complicated/conflicting, mutual assistance, love or strong attachment, confidence, explosive, no taboo…) | | 2 | 8 | *"And then, our relationship is... how can I say... not real. Not real, you know. Well, I don't really tell her anything about my life - I mean, just the most superficial things. Because, she doesn't really care, so, in reality, there's no point in telling someone who doesn't care about the real personal things." (PWA8)* |
