# Supplementary material for: The experience of albinism in France: a qualitative study on dyads of parents and their adult child with albinism
Source: BMC Med. 2024 Jan 29;22:40. doi: 10.1186/s12916-024-03251-z (PMC10823752; doi:10.1186/s12916-024-03251-z)
Supplement: Supplementary file 4 — Additional file 4. Health stigma and discrimination framework: This figure illustrates the model developed by Stangl et al. (2019), which seeks to synthesize the general mechanisms of stigma in a manner applicable to a diverse range of health-related issues. [file 12916_2024_3251_MOESM4_ESM.pdf]

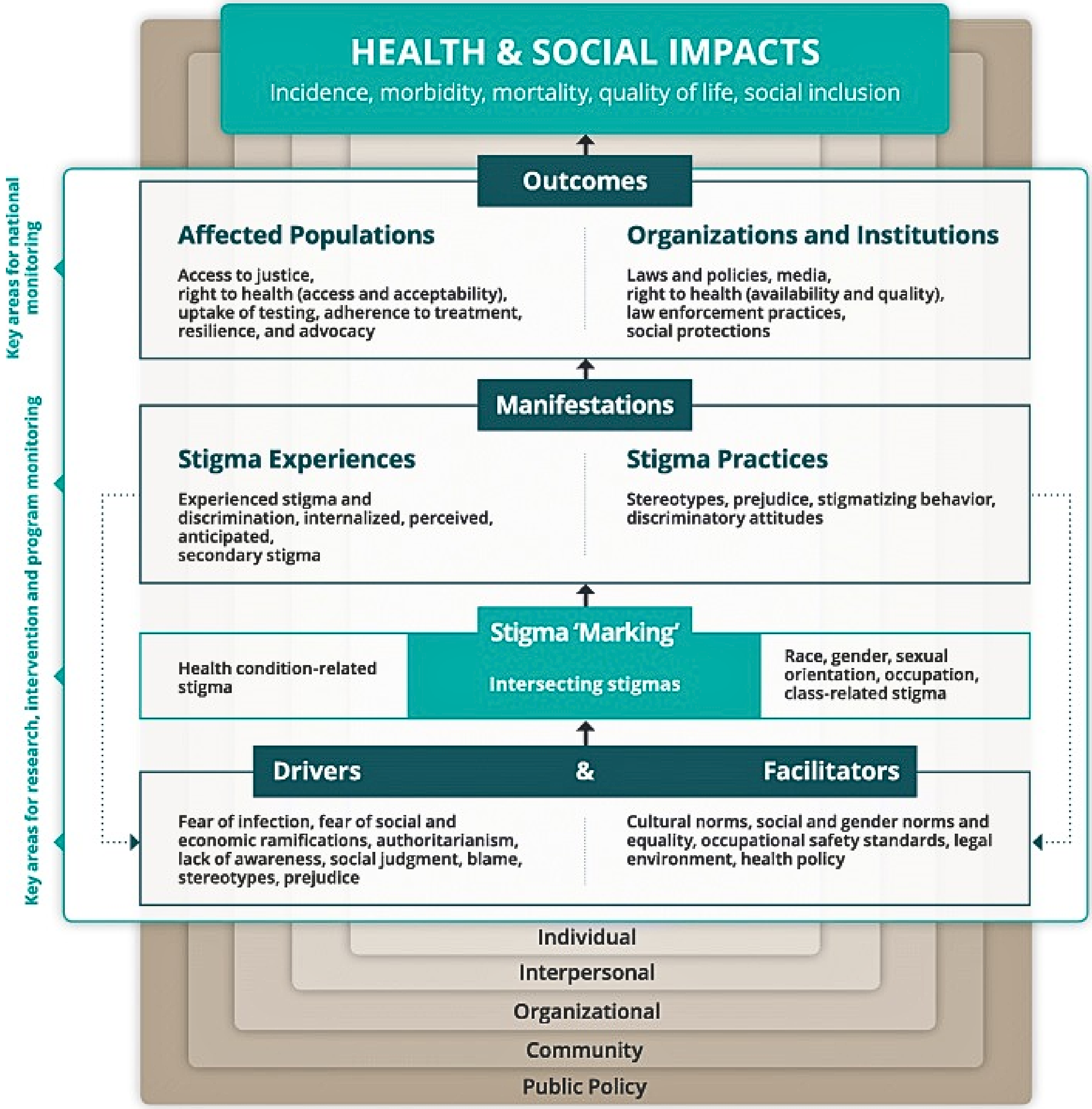

Stangl AL, Earnshaw VA, Logie CH, et al. The Health Stigma and Discrimination Framework: a global, crosscutting framework to inform research, intervention development, and policy on health-related stigmas. BMC Med. 2019;17(1):31. https://doi.org/10.1186/s12916-019-1271-3
